# Biomaterial-based strategies for postoperative residual tumors: From margin clearance to immune control and tissue repair

**DOI:** 10.1016/j.mtbio.2026.103167

**Published:** 2026-05-02

**Authors:** Meiyan Zou, Xu Chen, Nina Li, Zihao Zhou, Weiyao Feng, Rongwei Xu, Xinyuan Zhao, Li Cui

**Affiliations:** a Stomatological Hospital, School of Stomatology, Southern Medical University, Guangzhou, Guangdong, 510280, China; b School of Dentistry, University of California, Los Angeles, Los Angeles, CA, 90095, USA

**Keywords:** Residual tumor, Nanomaterial, Localized drug delivery, Immunogenic cell death, Tumor microenvironment, Cancer recurrence

## Abstract

Postoperative and post-ablation residual tumors represent a major yet underrecognized source of cancer recurrence, metastasis, and therapeutic failure. Distinct from primary tumors, residual lesions are typically sparse, spatially dispersed, and embedded within dynamically evolving microenvironments shaped by wound healing, inflammation, hypoxia, and immune imbalance. These features create pronounced spatiotemporal blind spots that limit the effectiveness of conventional systemic therapies and necessitate treatment strategies capable of precise local intervention, coordinated multimodal action, and active microenvironmental reconstruction. This review examines recent advances in nanomaterial-based approaches for the management of postoperative residual tumors, encompassing intraoperative and early postoperative nanotechnologies for high-resolution margin visualization and immediate disease clearance, as well as localized and sustained delivery systems—including hydrogels, implantable scaffolds, and microenvironment-responsive nanoparticles—that overcome spatial inaccessibility and temporal mismatch through prolonged retention and controlled activation. Multifunctional nanoplatforms integrating physical ablation, chemical or metabolic intervention, immune modulation, and tissue-supportive architectures are further highlighted for their ability to induce immunogenic cell death, remodel immunosuppressive niches, and concurrently promote postoperative tissue repair and functional recovery. Key challenges limiting clinical translation, including long-term biosafety, residual tumor heterogeneity, incomplete mechanistic resolution of immune effects, and the lack of standardized evaluation frameworks, are also discussed. By framing residual tumors as a coupled problem of tumor eradication and postoperative microenvironmental reconstruction, this review provides a unified conceptual framework to guide the rational design of nanomaterials for durable control of residual disease.

## Graphical abstract

**Figure:**
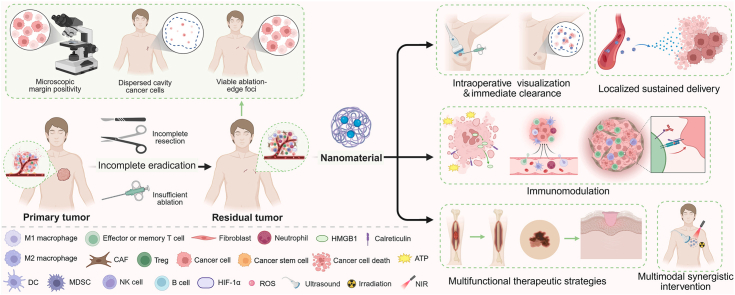

## 1 Introduction

Despite advances in early diagnosis and therapeutic strategies, cancer remains a leading cause of morbidity and mortality worldwide [1]. Surgical resection [2,3] and local ablative interventions—including radiotherapy [4], radiofrequency [5,6], microwave [7,8], and photothermal therapy (PTT) [9]—form the foundation of solid tumor treatment, yet complete eradication of malignant tissue is often challenging. Complex anatomical structures [10], infiltrative tumor growth [11], proximity to critical organs [12], and limitations in intraoperative imaging [13] frequently lead to incomplete resection or insufficient ablation, leaving microscopic or occult residual tumors. These residual tumors include microscopic disease at resection margins [14], dispersed tumor cells or microclusters within postoperative cavities, and viable malignant cells at ablation edges [15], rather than minimal residual disease, which more commonly refers to occult tumor burden detectable only by highly sensitive assays.

**Fig. 1:**
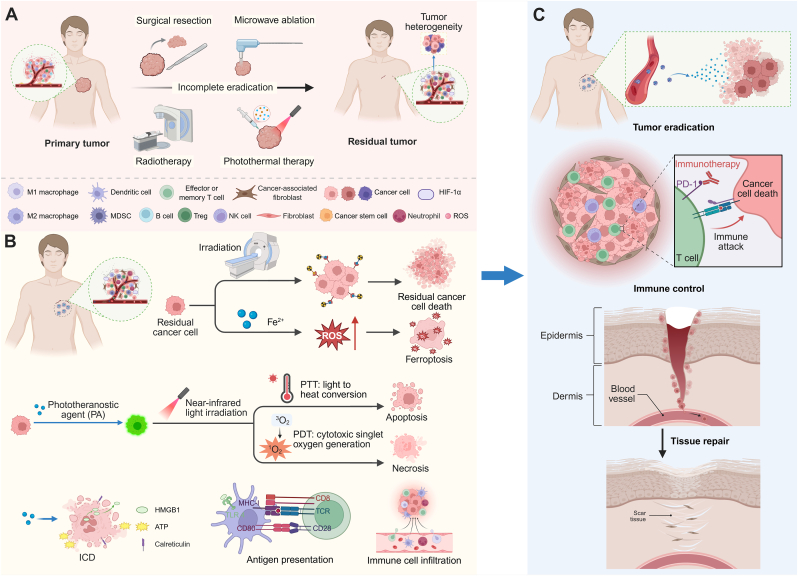
**Nanomaterial-based strategies for targeting postoperative residual tumors.** (A) Surgical resection and local ablative therapies, such as radiotherapy, microwave ablation, and PTT, often fail to fully eliminate primary tumors, resulting in residual tumor lesions characterized by sparse distribution, hypoxia, stromal remodeling, and diverse immune cell infiltration. (B) Nanomaterials enhance local tumor targeting by generating ROS, inducing ferroptosis, apoptosis, and necrosis, and triggering ICD, marked by CRT exposure, ATP release, and HMGB1. These events activate DCs, promoting antigen presentation and immune cell infiltration into the residual tumor. (C) Nanoplatforms synergize with ICB, enhancing T cell–mediated immune responses to eradicate residual tumors while supporting tissue repair and wound healing post-treatment.

Residual tumors should not be regarded as passive remnants of the primary lesion, but as treatment-perturbed niches undergoing active biological reprogramming [16]. Following regression or incomplete local treatment, residual tumor cells exhibit a distinct adaptive biology characterized by transcriptomic and metabolic rewiring, including glycolytic bias, urea-cycle alterations, lipid/reactive oxygen species (ROS) remodeling, and persistence of a tumor-like metabolic memory [17,18]. Sublethal thermal stress can further enrich stem-like and metastatic traits, including vascular endothelial growth factor receptor 1-dependent stemness programs [19], whereas insufficient ablation may directly promote epithelial mesenchymal transition-associated invasion through acute transforming retrovirus thymoma kinase (AKT)/extracellular signal-regulated kinase (ERK) signaling [20]. In parallel, the post-treatment microenvironment shifts toward immune escape, with expansion of myeloid-derived suppressor cells (MDSCs) and other suppressive myeloid populations [21], together with cancer-associated fibroblast (CAF)-mediated stromal-immune crosstalk that reinforces immunosuppressive cytokine, metabolic, and checkpoint-related programs [22] (Fig. 1A). To make these distinctions clearer, we have added a comparative table summarizing key biological differences among primary tumors, metastatic lesions, and residual tumors (Table 1). Following surgery or local ablation, the tumor microenvironment undergoes dynamic, stage-dependent changes that define distinct therapeutic windows for intervention. In the acute phase, representing an early inflammatory window, surgical trauma induces heightened inflammation [38,39] and hypoxia [40], creating conditions that may favor the survival and expansion of residual tumor cells [41]; at this stage, nanomaterials delivering anti-inflammatory or antioxidant agents may help mitigate early inflammatory injury and limit recurrence [42]. During the subsequent subacute repair phase, an immune reprogramming window emerges, characterized by extracellular matrix (ECM) remodeling [43] and shifts in immune composition [44], while surviving tumor cells acquire increased plasticity, stem-like features, and therapy resistance [45]; in this context, nanoparticles (NPs) releasing anti-fibrotic agents or immune adjuvants may help normalize matrix remodeling and enhance immune infiltration [46]. In the later stage, a vascular and stromal remodeling window is established, with persistent wound-healing signals, extensive stromal alterations, and immunosuppressive signaling that support tumor regeneration and progression [[47], [48], [49]]; targeted nanoplatforms that modulate macrophage polarization or tumor vasculature may help restrain disease progression and relapse [50]. Collectively, these stage-dependent windows provide a conceptual framework for the rational timing and functional design of nanomaterial-based interventions in postoperative residual tumor management.

**Table 1:** Comparison of primary tumors, metastatic lesions, and residual tumors across key microenvironmental and biological features.

Feature		Primary tumor VS metastatic lesion	Primary tumor VS residual tumor
Metabolism [17,18,[23], [24], [25]]	Glucose metabolism	Glycolysis rewiring ↑; lactate-associated adaptation ↑	Glycolysis ↑
	Lipid metabolism	Fatty acid uptake/oxidation ↑; lipid reprogramming ↑	Fatty acid metabolism ↑; β-oxidation ↑; lipid droplet accumulation ↑
	Amino acid metabolism	Glutamine dependence/glutaminolysis ↑	Urea-cycle rewiring ↑; arginine uptake ↑; glutamine uptake ↑
	Oxidative metabolism	Oxidative phosphorylation plasticity ↑; tricarboxylic acid cycle rewiring ↑	Oxidative phosphorylation flexibility ↓; hypoxia–autophagy coupling ↑
Immune infiltration [[26], [27], [28], [29], [30], [31]]	T cells	CD8^+^ T cells often ↓; Treg enrichment ↑; T cell exhaustion ↑	CD8^+^/CD4^+^ T cell function ↓; Treg accumulation ↑; PD-1/PD-L1-associated exhaustion ↑
	Natural killer (NK) cells	NK cell cytotoxicity often ↓	NK cell activity ↓
	Tumor-associated macrophages (TAMs)/monocytes	TAM infiltration ↑; M2-like polarization ↑	Monocyte recruitment ↑; TAM accumulation ↑; M2-like polarization ↑
	Neutrophils/MDSCs	Neutrophil recruitment ↑; MDSC enrichment ↑	Acute neutrophil infiltration ↑; MDSCs ↑
	Dendritic cells (DCs)	DC maturation/antigen presentation often ↓	DC maturation/antigen presentation ↓
ECM stiffness [27,31,32]		Desmoplasia often ↑; stiffness altered in a site-dependent manner	Matrix stiffness ↑; wound-like fibrotic niche ↑
Vascularization [26,[33], [34], [35]]		Angiogenesis often ↑; vessel co-option may ↑ in some lesions	Re-angiogenesis ↑; microvessel density ↑
ROS level [17,23,36,37]		Oxidative-stress adaptation ↑; antioxidant buffering ↑	Post-treatment ROS surge ↑; oxidative DNA damage ↑; redox adaptation/antioxidant compensation ↑

Current guideline-based management of postoperative residual or locoregionally recurrent tumors still relies mainly on repeat local treatment and risk-adapted adjuvant therapy. In breast cancer, postmastectomy radiotherapy remains an important option for selected patients with residual disease or positive margins [51], but its benefit is limited by dose constraints and late local toxicity such as fibrosis and edema [52]. In head and neck squamous cell carcinoma and recurrent nasopharyngeal carcinoma, salvage surgery or re-irradiation may be considered for selected locoregional relapse [53,54], yet durable control is often restricted by anatomical complexity, prior treatment, and toxicity to adjacent critical structures [55,56]. In hepatocellular carcinoma (HCC), repeat resection or ablation is preferred when feasible, whereas stereotactic radiotherapy, proton therapy, or brachytherapy may be used after incomplete or recurrent local treatment [57], although retreatment is frequently limited by liver reserve, tumor location, and suboptimal margin control [58]. More broadly, these approaches may still leave poorly perfused postoperative cavities or ablation margins insufficiently covered [59,60], while dose escalation is limited by systemic toxicity and the risk of impaired wound healing [54]. Their efficacy is also undermined by the temporal and spatial mismatch with the evolving biology of residual tumors, together with postoperative hypoxia, immune suppression, and the high adaptability of surviving cells [44,61,62].

In this context, nanomaterials, with their nanoscale properties and high design flexibility, offer new avenues for precision treatment of postoperative residual tumors. By tuning particle size, surface chemistry, and structural morphology, nanomaterials can selectively accumulate in hard-to-reach postoperative regions through the synergistic effects of enhanced permeation and retention and active targeting, thereby improving local delivery efficiency while minimizing systemic exposure [63,64]. Their modular and multifunctional nature allows integration of physical therapies, drug delivery, and immune modulation to achieve coordinated multimodal intervention [65] (Fig. 1B). Nanoplatforms can also be engineered to respond to signals within the postoperative microenvironment, precisely activating therapeutic effects during the window when residual tumor cells are most vulnerable, thereby addressing the spatiotemporal mismatch of conventional therapies [66,67]. Simultaneously, nanomaterials can induce immunogenic cell death (ICD), enhance antigen presentation, and promote immune cell infiltration to remodel the local immunosuppressive environment, partially converting local tumor clearance into systemic immune protection [68,69]; some nanomaterials also support tissue repair and functional restoration [[70], [71], [72]] (Fig. 1C). Despite progress, current studies still lack systematic integration and a holistic understanding of these strategies.

In this review, we systematically summarize advances in nanomaterial-based therapies for postoperative residual tumors, focusing on three main areas: first, intraoperative precision visualization and immediate clearance strategies, highlighting design principles for high-resolution tumor margin identification and in situ ablation; second, local sustained delivery and multimodal synergistic strategies, illustrating how long-term accumulation, spatiotemporally precise release, and physical–chemical–immune synergy overcome microenvironment complexity and therapy resistance; and third, nanomaterial-driven immune modulation and multifunctional therapeutic strategies, detailing mechanisms by which they induce ICD, remodel immunosuppressive microenvironments, and synergize with immune checkpoint blockade (ICB) to establish durable memory, while integrating tumor eradication, tissue repair, and infection control to form a unified framework spanning local intervention, immune reprogramming, and functional recovery. Finally, we highlight the major challenges that currently limit nanomaterial-based strategies for postoperative residual tumor management and discuss future directions ranging from rational material design to system-level precision interventions, aiming to provide theoretical guidance and technical insights for the precision treatment and long-term management of postoperative residual tumors.

## 2 Intraoperative and postoperative nanomaterial strategies for residual tumor recognition and immediate clearance

Incomplete surgical resection is a major cause of tumor recurrence and poor patient outcomes [73]. Recent advances in nanomaterials have enabled precise intraoperative visualization of residual tumor margins and immediate postoperative elimination of residual tumor cells [74,75]. By combining imaging, therapeutic, and immunomodulatory functions, these nanoplatforms not only improve the precision of surgical resection but also remodel the local immune microenvironment to prevent recurrence [76].

### 2.1 Precise intraoperative visualization of residual tumor margins

**Fig. 2:**
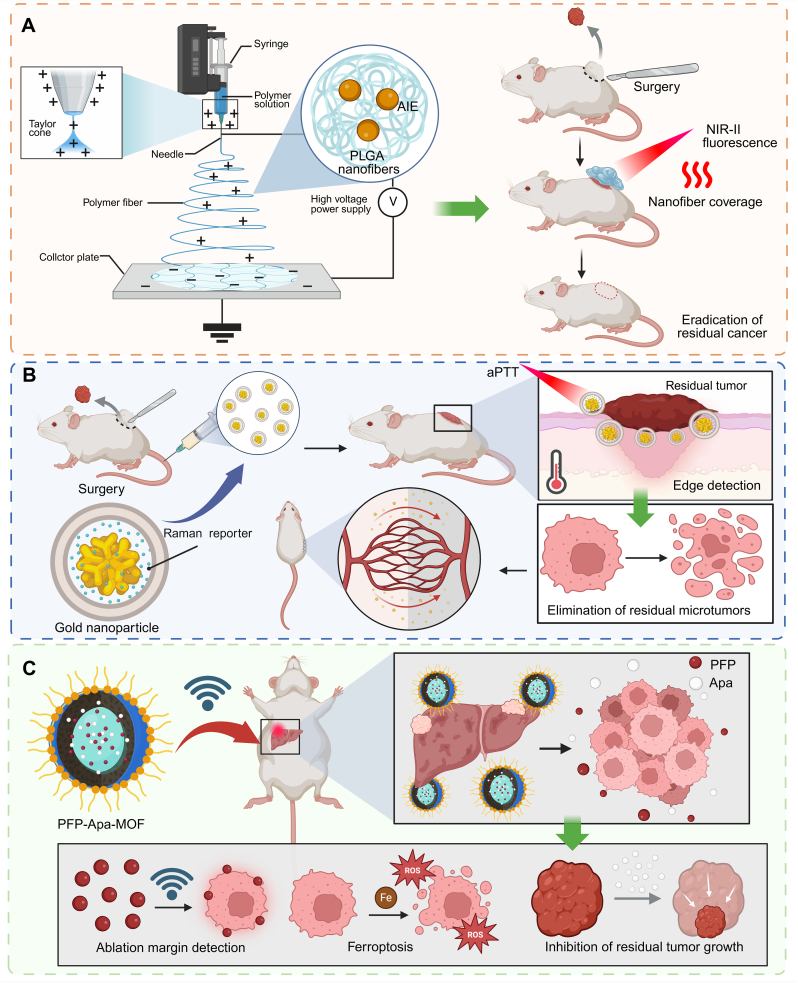
**Nanomaterial-based strategies for immediate clearance and recognition of residual tumors during and after surgery.** (A) An AIE-functionalized PLGA nanofiber scaffold, implantable at tumor resection margins, enables sensitive NIR-II fluorescence visualization of residual microtumors. It also provides repeated on-demand PTT under external NIR irradiation, achieving localized elimination of residual cancer cells. (B) SERS-optimized gold nanoparticles facilitate precise localization of residual tumor boundaries and enable ablation of microtumors through NIR laser-induced aPTT. The platform demonstrates prolonged blood circulation, high tumor accumulation, and effective tumor clearance. (C) PFP-Apa-MOF NPs facilitate ferroptosis induction via iron release, in which PFP serves as an ultrasound contrast agent for ablation margin detection, while residual tumor growth is inhibited through Apa-mediated effects. (For interpretation of the references to color in this figure legend, the reader is referred to the Web version of this article.)

Fluorescence-guided surgery (FGS) improves intraoperative tumor visualization, but its sensitivity for microscopic residual lesions and its ability to provide immediate local treatment remain limited [77]. In this context, nanomaterials can enhance FGS by improving signal performance and enabling integrated theranostic intervention. or example, A1 NPs, an aggregation-induced emission (AIE)-active near-infrared (NIR)-II phototheranostic, enabled precise intraoperative detection of microscopic residual tumors as well as metastatic lymph nodes. More importantly, this platform combined NIR-II fluorescence-guided identification with immediate phototherapy of unresectable residual lesions, thereby achieving complete tumor eradication without postoperative local recurrence or metastasis [78]. Similarly, electrospun poly(lactic-co-glycolic acid) (PLGA) nanofibers incorporating AIE luminogens (AIEgens) form a biodegradable patch that integrates NIR-II fluorescence imaging with photothermal activity. Implanted at surgical resection margins, the nanofiber scaffold enables sensitive visualization of residual microtumors and supports repeated, on-demand photothermal ablation (PTA) under external irradiation, achieving localized elimination of residual cancer cells while minimizing systemic exposure [79] (Fig. 2A). In addition, gold nanoparticles optimized for surface-enhanced Raman scattering imaging were developed for intraoperative tumor margin detection and PTA. These nanoparticles allowed precise localization of tumor boundaries and ablation of residual micro-tumors using NIR laser-induced adjuvant photothermaltherapy (aPTT). In vivo, the nanoparticles exhibited prolonged blood circulation, high tumor accumulation, and no toxicity, significantly prolonging survival and delaying tumor recurrence in mouse models [80] (Fig. 2B).

### 2.2 Immediate physical ablation of residual tumors enabled by nanomaterials

Postoperative or intraoperative ablation strategies using nanomaterials integrate physical and catalytic mechanisms to eliminate residual tumor cells while promoting immune activation. A two-photon excitable photosensitizer (MeTTh) was developed for precise treatment of small glioblastomas (GBMs) using NIR-II light. MeTTh integrates multiple design features, including large absorption cross-section, NIR-I emission, and efficient Type I/II ROS generation. Nanofabricated MeTTh NPs enabled deep brain imaging up to 940 μm and precise treatment of small-size GBM in vivo under 1040 nm fs laser irradiation [81]. Furthermore, an iron-based metal-organic framework nanomedicine (PFP-Apa-MOF) was developed for enhancing microwave ablation (MWA) therapy in HCC. The nanomedicine, loaded with perfluoropentane (PFP) and apatinib (Apa), induces ferroptosis via iron release, acts as an ultrasound agent for ablation margin detection, and inhibits residual tumor growth with Apa. In vitro and in vivo, MWA combined with PFP-Apa-MOF significantly enhanced ablation efficiency, promoting tumor apoptosis, lipid peroxidation, and improved therapeutic outcomes [82] (Fig. 2C).

## 3 Localized and sustained postoperative therapy mediated by nanomaterial delivery systems

While intraoperative and immediate postoperative nanomaterial strategies aim to identify and eliminate residual tumor cells at the time of surgery, microscopic disease may persist beyond this early window and continue to drive recurrence. Sustained and localized drug delivery to the surgical cavity therefore represents an important postoperative strategy for maintaining therapeutic exposure at the resection site and prolonging control over residual tumor cells. However, effective delivery in the postoperative setting remains highly challenging because surgery or local ablation profoundly reshapes transport routes, vascular supply, and the stromal architecture of residual tumor niches. Prior local treatment can alter lymphatic drainage patterns, redirecting flow away from conventional pathways and rendering postoperative transport and regional distribution of therapeutic agents less predictable [83]. At the same time, local perfusion is often reduced after ablation, whereas incompletely ablated lesions may retain relatively higher peri-tumoral perfusion, highlighting pronounced spatial heterogeneity in blood supply within the post-treatment microenvironment [84]. In parallel, the residual tumor microenvironment may become increasingly restrictive due to ECM remodeling, matrix densification, and fibrosis barrier formation. In residual melanoma, therapy-induced ECM remodeling creates a transient immune barrier that spatially excludes CD8^+^ T cells [43]. Similarly, in residual HCC following suboptimal heat treatment, collagen I accumulation at the periablational margin promotes aggressive behavior of surviving tumor cells [85]. Collectively, these alterations in lymphatic routing, vascular perfusion, matrix density, and fibrotic remodeling can substantially impair nanomedicine penetration, distribution, retention, and sustained therapeutic efficacy in postoperative residual tumors. In this context, nanomaterial-based hydrogels, implantable scaffolds, and slow-release nanoparticle formulations offer a means to achieve precise, spatiotemporally controlled local drug release, thereby enhancing therapeutic persistence at the surgical site while minimizing systemic toxicity.

### 3.1 Localized nanohydrogel delivery systems

Postoperative nanohydrogels integrate nanomedicines within injectable or sprayable hydrogel matrices, forming surgical cavity–conforming drug depots that enhance local drug retention, spatial precision, and sustained therapeutic exposure at resection margins [86].

#### 3.1.1 Stimuli-responsive nanohydrogels

Stimuli-responsive nanohydrogels exploit physiological cues or externally applied energy to trigger on-demand drug release and therapeutic activation [87]. This conditional responsiveness allows drug release to be temporally aligned with postoperative residual disease dynamics. In bone tumor–associated settings, injectable hydrogels have been engineered to couple controlled drug release with physical or catalytic therapies. A doxorubicin (DOX)-loaded magnetic alginate hydrogel (DOX@MAH), incorporating iron oxide nanoparticles for magnetic hyperthermia, exhibited high drug-loading capacity, continuous DOX release, and effective tumor elimination under an alternating magnetic field. The hydrogel formed in situ upon contact with calcium ions in bone tissue, ensuring intimate adaptation to surgical defects. In vivo, DOX@MAH significantly inhibited tumor growth and induced extensive necrosis, highlighting its potential to prevent postoperative recurrence and metastasis [88].

**Fig. 3:**
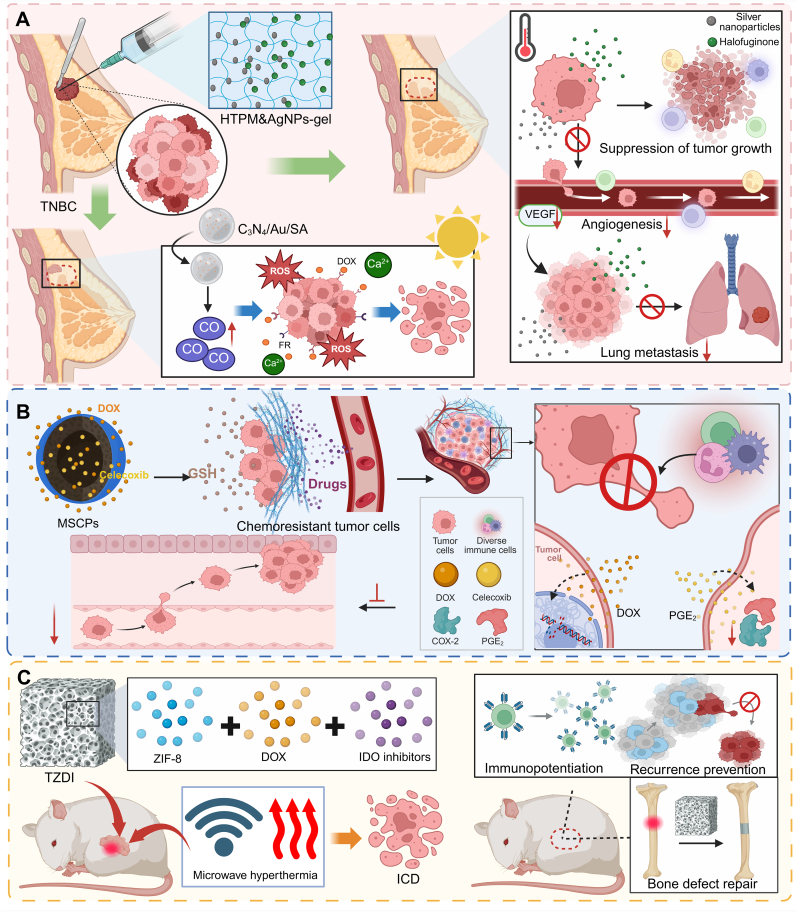
**Nanomaterial-based strategies for postoperative tumor control and therapy.** (A) HTPM&AgNPs-gel, a thermosensitive hydrogel with halofuginone and AgNPs, inhibits TNBC growth, lung metastasis, and angiogenesis. Sodium alginate hydrogel with Au@g-C_3_N_4_ nanophotocatalysts, applied at postoperative margins, generates CO under visible light to enhance oxidative stress, amplifying the efficacy of folate-DOX micelles. Ca^2+^-triggered gelation traps nanodrugs at the resection site, improving precision and reducing systemic toxicity. (B) Redox-responsive MSCPs co-loaded with DOX and celecoxib deliver both agents efficiently, enabling celecoxib to inhibit COX-2/PGE_2_ signaling, suppress chemotherapy-induced cancer stemness and invasiveness, and enhance DOX's antitumor activity, thereby preventing tumor repopulation, metastasis, and drug resistance. (C) TZDI scaffolds, combining microwave-responsive ZIF-8 nanomaterials with 3D-printed Ti-scaffolds, induce tumor ICD via microwave-triggered hyperthermia and chemotherapy, enhancing antitumor immunity and eliminating residual bone tumor cells to prevent recurrence.

Beyond bone tumors, stimuli-responsive hydrogels have been applied to solid tumors requiring precise postoperative modulation of the immune microenvironment. In triple-negative breast cancer (TNBC), a thermosensitive hydrogel loaded with halofuginone and silver nanoparticles (HTPM&AgNPs-gel) exhibited syringeability, swelling capacity, and sustained release without hemolysis, effectively inhibiting tumor proliferation, migration, invasion, and angiogenesis in vitro and markedly suppressing tumor growth, lung metastasis, and angiogenesis in vivo [89] (Fig. 3A). In addition to TNBC-targeted hydrogels, postoperative breast cancer margins have been treated using sodium alginate hydrogels incorporating Au-decorated g-C_3_N_4_ nanophotocatalysts (C_3_N_4_/Au/SA). Under visible light, these photocatalysts generate carbon monoxide in situ, increasing intratumoral oxidative stress and synergistically enhancing folic acid-modified DOX micelle-mediated chemotherapy, while rapid Ca^2+^-induced gelation helps retain the nanomedicine at the resection site and reduce systemic toxicity. This approach highlights how local catalytic activation can complement chemotherapeutic delivery within a hydrogel platform for effective residual tumor clearance [90] (Fig. 3A). Stimuli-responsive hydrogels can further integrate multimodal therapeutic functions. An injectable hydrogel composed of dopamine-modified polymer networks and MnO_2_ nanocomponents (NDP@MnO_2_) achieved conformal coverage of pancreatic tumor resection margins and enabled localized PTA under 808 nm irradiation, efficiently eliminating residual tumor cells while minimizing collateral damage [91]. Similarly, a curcumin (Cur)-loaded hollow mesoporous organosilica hydrogel (Cur@HMON@gel) allowed sustained curcumin release and ultrasound-triggered sonodynamic therapy (SDT), effectively reducing blood perfusion in residual renal cell carcinoma (RCC) after ablation [92].

#### 3.1.2 Self-programmed nanohydrogels

In contrast to stimuli-responsive nanohydrogels, self-programmed nanohydrogels achieve prolonged postoperative therapy at surgical sites through intrinsic material properties, such as slow matrix degradation, pH-responsive coordination chemistry, or diffusion-controlled release, without requiring external activation [93]. For example, a sprayable hyaluronic acid hydrogel embedding polydopamine-coated oxaliplatin (OXA)-Mn^2+^-Cur coordination polymer nanoparticles (ICP@PDA NPs@composite hydrogel) provided strong tissue adhesion and pH-responsive, multi-stage drug release for over 20 days, driving coordinated chemotherapy and immunoactivation. Local application eradicated residual tumors, completely suppressed peritoneal metastasis, and achieved long-term recurrence-free survival with minimal systemic toxicity [94].

Self-programmed hydrogels have also been leveraged to deliver immunomodulatory nanomedicines in residual tumors. In HCC after incomplete radiofrequency ablation (iRFA), a poly(d,L-lactide)-poly(ethylene glycol)-poly(d,L-lactide) (PDLLA-PEG-PDLLA) nanocomposite hydrogel incorporating TAK-981 (a SUMOylation inhibitor) and bovine serum albumin (BSA) nanoparticles achieved sustained local inhibition of SUMOylation (BT-NPs@PLEL nanocomposite hydrogel), enhancing DC maturation and cytotoxic lymphocyte–mediated antitumor responses. When combined with PD-L1 blockade, this strategy effectively eradicated both residual and distant tumors [95]. In breast cancer, an implantable photo-responsive self-healing hydrogel loaded with MoS_2_ nanosheets and the immunoadjuvant R837 (PVA-MoS_2_-R837) enabled localized PTA and in situ immune activation, significantly suppressing postoperative recurrence when combined with immune checkpoint inhibition. In brain tumors, where postoperative recurrence is driven by infiltrative growth and limited drug penetration, self-programmed hydrogels provide durable local therapeutic coverage at the resection site [96]. Beyond immune regulation, hydrogels can also provide prolonged chemotherapeutic delivery. In GBM, an injectable lipid nanocapsule–based hydrogel loaded with lauroyl-DOX prodrug (DOXC_12_-LNC^CL^) demonstrated sustained drug release for over one month, with high encapsulation efficiency and potent in vitro and in vivo efficacy, delaying tumor recurrence when combined with anti-inflammatory treatment [97]. Similarly, a dual-sensitive but self-programmed hydrogel system combining ROS-sensitive PLGA NPs loaded with bis(2-chloroethyl) nitrosourea (BCNU) and temozolomide (TMZ) together with free drugs (BCNU-TMZ&ROS NPs@Gel) enabled long-term release, achieving significant inhibition of glioma recurrence and extending median survival after >90% tumor resection [98]. Additional hydrogel platforms have also been explored for postoperative local delivery in glioma. A bioadhesive pectin hydrogel carrying polylactic acid-polyethylene glycol-coated drug nanocrystals (NCPPs) was developed for use immediately after surgery at a stage of low residual tumor burden, enabling stable etoposide and olaparib release over 120 h and efficient local delivery to brain tissue [99]. In parallel, a thermosensitive PLGA-PEG-PLGA hydrogel co-encapsulating glioma-homing paclitaxel (PNP_PTX_) and mannitolated CpG immunoadjuvant (MNP_CpG_) NPs formed a sustained-release depot in the resection cavity. The PNP_PTX_&MNP_CpG_ hydrogel inhibited residual glioma cells and activated antigen-presenting cells, thereby enhancing CD8^+^ T and NK cell-mediated immunity and improving therapeutic efficacy through combined chemo-immunotherapy [100].

Beyond local tumor control, self-programmed hydrogels can generate systemic therapeutic benefits. Co-delivery of Cur-loaded nanomedicine and antigenic peptide/CpG-ODN nanovaccine via thermo-responsive hydrogels induced ICD and DC activation, enhancing CD8^+^ T cell-mediated antitumor immunity and suppressing recurrence and metastasis in postoperative 4T1 breast carcinoma models [101]. In combination with systemic therapy, ROS-scavenging gels loaded with anticancer drugs, together with albumin nanoparticle-mediated clopidegrol delivery, inhibited residual microtumor growth and lung metastasis [102]. As an additional demonstration, MRI-traceable ultra-thermosensitive hydrogels co-delivering epirubicin and paclitaxel provided prolonged, localized chemotherapy, markedly extending survival in orthotopic gliosarcoma models and achieving durable suppression of recurrence [103].

### 3.2 Local sustained-release nanoparticle formulations

Nanoparticles provide sustained and localized delivery of therapeutic agents to residual tumors, improving tumor-site accumulation, tissue penetration, and selective targeting. Their modular design enables co-delivery of multiple drugs and functionalization for immune modulation, enhancing antitumor efficacy while reducing systemic toxicity [104].

#### 3.2.1 Microenvironment-responsive nanoparticles

Microenvironment-responsive nanoparticles leverage tumor-specific microenvironmental cues such as hypoxia, necrotic regions, redox imbalance, lysosomal acidity, and elevated enzymatic activity to trigger selective drug release and enhance antitumor efficacy [105,106]. Hypoxia-activated prodrug nanoparticles assembled from an azobenzene-linked TLR7/8 agonist (Azo-IMDQ-NPs) selectively release imidazoquinoline (IMDQ) within the hypoxic microenvironment created by iRFA. Local activation reprograms immunosuppressive tumor niches, amplifies ablation-induced antitumor immunity, and eliminates residual HCC, while establishing durable immune memory that suppresses postoperative recurrence [107]. Beyond hypoxia, a biodegradable nanoparticle (PNP@(^131^I-Hyp)) targeting necrosis was developed for residual tumor treatment following thermal ablation of HCC. The nanoparticle, composed of iodine-131-labeled hypericin (^131^I-Hyp) as the core and PEG-block-poly(ε-caprolactone) (PEG-PCL) copolymer as the shell, exhibited uniform spherical morphology and excellent stability. In vivo, it accumulated in necrotic tumor zones, as confirmed by fluorescence and single photon emission computed tomography (SPECT) imaging, and effectively inhibited residual tumor growth via radiotherapy [108]. Celecoxib, a COX-2 inhibitor, enabled MSCPs to inhibit COX-2/PGE_2_ signaling, which normally promotes tumor repopulation and invasiveness. poly(β-cyclodextrin) wrapping (MSCPs) efficiently co-delivered DOX and celecoxib to the tumor, enhancing DOX's antitumor activity while suppressing cancer stemness and invasiveness. In preclinical models, the DOX@MSCPs system effectively prevented tumor repopulation, metastasis, and drug resistance, demonstrating promising potential for improving chemotherapy outcomes [109] (Fig. 3B). Delving into intracellular release, a lysosomal-targeting self-condensation prodrug-nanoplatform (LTSPN) was developed using hydroxycamptothecine (HCPT)-silane conjugates that self-assembled into nanoparticles. Upon lysosomal uptake, acid-triggered hydrolytic condensation destabilized the lysosomal membrane, releasing alcohol and transforming nanoparticles into silicon particles. The LTSPN system enhanced the effectiveness of HCPT, reducing its IC_50_ by four times. It also improved control over large tumors and inhibited the regrowth of residual drug-resistant tumors, offering a promising strategy for overcoming chemoresistance in malignancies [110]. Furthermore, an enzymatically transformable polymer-based nanotherapeutic (ETP) approach targets postsurgical residual cancer by exploiting matrix metalloproteinase (MMP) overactivation. The system designated as Col/M@ETP co-delivers colchicine (microtubule-disrupting and anti-inflammatory agent) and marimastat (MMP inhibitor), with MMP-driven dePEGylation enhancing tissue targeting and retention. The nanoparticles selectively release marimastat in extracellular spaces and colchicine intracellularly, disrupting both the premetastatic microenvironment and microtubules. This approach reduces metastatic and local-regional recurrence by effectively targeting microresiduals and the premetastatic niche after surgery, improving the therapeutic outcomes in metastatic breast cancer [111]. Collectively, these microenvironment-sensitive nanoparticles demonstrate precise, localized therapeutic control and the ability to overcome resistance mechanisms.

#### 3.2.2 Biomimetic and cell-targeted nanoparticles

Biomimetic and cell-targeted nanoparticles enhance postoperative tumor-site accumulation and therapeutic specificity by leveraging cell membrane camouflage or receptor-mediated targeting [112]. Immortalized mesenchymal stem cell plasma membranes were used to fabricate biomimetic nanoparticles encapsulating shCD73 and DOX (Lipo-PM@shCD73@DOX). These membrane-camouflaged nanomedicines efficiently crossed the blood-brain barrier (BBB) and selectively accumulated in postoperative residual glioma. Targeted delivery enabled combined gene silencing and chemotherapy, resulting in suppressed tumor cell proliferation, enhanced apoptosis, and delayed glioma progression, while maintaining favorable in vitro and in vivo biosafety profiles [113]. Similarly, a platelet membrane-derived nanovesicle (PMVS-P) loaded with salinomycin (SAL) and surface-functionalized with polydopamine targets glioblastoma stem cells (GSCs) via the D2 dopamine receptor, which is highly expressed on GSCs at surgical margins. The nanovesicle effectively inhibits GBM recurrence by delivering GSC-specific tumoricidal treatment, leveraging its surgical incision targeting ability [114]. In parallel, a novel NE-like membrane system (NM-PD) was developed by coating NE-like membranes onto PLGA-PEG-based DOX-loaded nanoparticles (PLGA-PEG-DOX). The NM-PD exhibited inflammatory chemotaxis similar to mature neutrophils, enhancing BBB permeability and anti-proliferative effects on GBM cells in vitro. In an intracranial GBM resection model, NM-PD targeted residual GBM cells, significantly improving antitumor activity and prolonging survival [115].

### 3.3 Implantable nanoscaffolds and fiber patches

Implantable nanoscaffolds and fibrous membranes provide structural support and functional versatility for postoperative cancer therapy, unlike soft hydrogels that mainly serve as drug depots. By mimicking the ECM and incorporating therapeutic nanomaterials, these implants can eradicate residual tumor cells [116].

In bone tumors, implantable scaffolds have been developed to eradicate residual tumor cells while providing localized drug delivery. For instance, multifunctional therapeutic scaffolds Ti-ZIF-8@DOX-IDO (TZDI) were fabricated by integrating microwave-responsive zeolitic imidazolate framework-8 (ZIF-8) nanomaterials, loaded with chemotherapeutic agents and immune checkpoint inhibitors, onto 3D-printed titanium scaffolds. These scaffolds induced immunogenic tumor cell death through microwave hyperthermia and chemotherapy and enhanced antitumor immune responses to prevent recurrence [117] (Fig. 3C). In breast tumors, scaffold-based drug delivery has been applied to achieve sustained chemotherapy in the postoperative tumor cavity. An injectable chitosan/polyethylene oxide electrospun fibrous scaffold co-delivering celecoxib (CEL) and DOX-loaded tumor cell-derived microparticles provided prolonged drug release and effectively eliminated residual tumor cells, while suppressing polymorphonuclear neutrophils (PMN) -mediated tumor proliferation, invasion, migration, and neutrophil extracellular trap formation [118]. Similar delivery concepts have also been extended to other solid tumors. In bladder cancer, multilayer electrospun mats enabled controlled, localized delivery of gemcitabine (GEM) and cisplatin (CDDP) to positive surgical margins, effectively inhibiting tumor cell growth, inducing apoptosis, and reducing liver toxicity compared with systemic chemotherapy [119] (Table 2, Supplementary Table 2).

**Table 2:** Representative localized nanomaterial-mediated delivery strategies for residual tumor therapy.

Nanomateial delivery system	Loaded drug	Release mechanism	Local delivery advantage	Tumor model	Therapeutic outcome	Ref.
DOX@MAH	DOX	Magnetically triggering hyperthermia-assisted sustained drug release	Achieving high drug loading and sustained release, forming in situ and conforming to bone defects	Post-resection bone tumor	Inducing extensive necrosis and 92.44% postoperative tumor inhibition	[88]
HTPM&AgNPs-gel	HTPM, AgNPs	Thermally regulating sustained drug release	Retaining at resection margins via syringeable hydrogel formation	Postoperative orthotopic TNBC	75.46% tumor inhibition; suppressing angiogenesis and lung metastasis	[89]
C_3_N_4_/Au/SA hydrogel	C_3_N_4_/Au	Converting CO_2_ within the tumor into CO under visible light irradiatio	Increasing the concentration of CO within the tumor and reducing systemic toxicity	Postoperative residual breast cancer	Enhancing chemotherapy efficacy by increasing oxidative stress	[90]
ICP@PDA NPs@composite hydrogel	OXA, Mn^2+^, Cur	pH-responsively coordinating multi-stage drug release	Providing strong tissue adhesion and prolonged local drug retention	Postoperative residual CRC	Reducing blood perfusion, 100% survival (80 d); intraperitoneal tumors eradicated in 20 d	[94]
BT-NPs@PLEL nanocomposite hydrogel	TAK-981	Sustaining release via slow hydrogel degradation	Achieving high local drug concentration and minimizing systemic toxicity	Residual HCC after RFA	Enhancing antitumor immunity and curbing the expansion of residual tumors	[95]
BCNU-TMZ&ROS NPs@Gel	BCNU, TMZ	ROS-sensitively regulating long-term multi-drug release	Maintaining high local drug concentration	Postoperative orthotopic GBM	Reducing tumor volume to 1.5% at day 35, prolonging median survival to 65 d	[98]
Azo-IMDQ-NPs	IMDQ	Hypoxia-triggering prodrug activation	Selectively releasing immunostimulant in hypoxic residual tumor sites, minimizing off-target toxicity	Residual HCC after iRFA	Reprogramming immunosuppressive TME, 87.9% tumor suppression, ↑median survival >60 d	[107]
Col/M@ETP	Colchicine, Marimastat	MMP-activating dePEGylation and dual-compartment release	Targeting postsurgical wounds and premetastatic niches, enhancing retention	Postsurgical residual breast tumor	∼60%/80% recurrence-free; >80% metastasis-free	[111]
Lipo-PM@shCD73@DOX	shCD73, DOX	Cell membrane–mediated targeting with intracellular release	Crossing BBB and accumulating in postoperative glioma sites	Postsurgical residual glioma	Delaying glioma progression by inhibiting cellular proliferation and inducing apoptosis	[113]
TZDI	IDO inhibitor, DOX	Degradation-mediated Zn^2+^ release and microwave-triggered drug release	Enabling confined drug accumulation at the tumor–bone interface	Orthotopic femur osteosarcoma	Suppressing tumor recurrence, inducing ICD, and promoting osteogenesis	[117]
CEL/DOX-MPs@CP	CEL, DOX	Degradation-controlled sustained release	Maintaining prolonged retention and in-situ delivery within resection cavities	Postsurgical orthotopic breast tumor	Eliminating residual tumor cells and suppressing neutrophil-mediated tumor progression	[118]
multi-GC@PLA mat	GEM, CDDP	Layer-controlled sustained drug release	Localizing chemotherapy to positive surgical margins	Postoperative positive-margin bladder cancer	83.6% tumor suppression, preventing local recurrence	[119]

## 4 Nanomaterial-mediated multimodal strategies

After surgery or local intervention, residual tumor foci are often small, scattered, and difficult to eradicate completely. At this stage, additional therapeutic reinforcement is often needed to eliminate microscopic residual disease that escapes initial treatment. Although physical modalities can destroy most visible lesions, microscopic tumor cells may persist beyond the effective treatment range, whereas chemotherapy and metabolic interventions are often limited by insufficient penetration, adaptive resistance, and the complexity of the post-treatment microenvironment. In this context, nanomaterials provide a versatile platform for integrating drugs, sensitizers, or other functional agents, thereby enabling complementary therapeutic effects and improving the depth and durability of residual tumor control.

### 4.1 Physical and phototherapeutic strategies

#### 4.1.1 Photothermal therapy

**Fig. 4:**
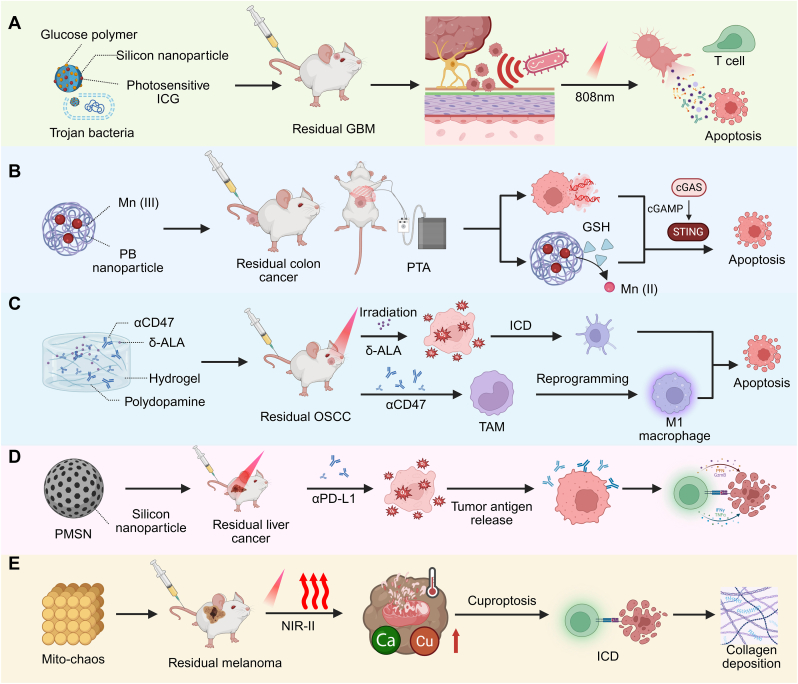
**Nanomaterial-mediated multimodal strategies for residual tumors.** (A) Trojan-bacteria delivery system for GBM therapy: glucose-polymer-coated silicon nanoparticles loaded with the photosensitizer ICG accumulate within the tumor under 808 nm laser irradiation, producing intense photothermal killing of cancer cells and concomitant T cell activation. (B) Mn (III)-doped Prussian blue nanoparticles mediate PTA, ignite the cGAS-STING pathway and boost innate immune signaling, promoting antitumor immunity against both local and distant colon cancer lesions. (C) δ-ALA-based PDT combined with polydopamine-augmented PTT evokes ICD, enhances DC activation and repolarizes TAMs toward the M1 phenotype for OSCC treatment. (D) Ultrasound-triggered PMSN generates ROS that oxidatively damage residual HCC cells; when combined with PD-L1 ICB this facilitates antigen release and robust immune activation. (E) Mito-chaos delivers Cu^2+^ and Ca^2+^ ions into tumor cell mitochondria, inducing cuproptosis via ionic homeostasis disruption that is further amplified by mild NIR-II photothermal heating; the platform simultaneously promotes collagen deposition to accelerate wound healing after surgery in residual melanoma. (For interpretation of the references to color in this figure legend, the reader is referred to the Web version of this article.)

PTT-based nanoplatforms represent the most direct and intuitive approach for eliminating postoperative residual tumors, relying on localized heat generation under external irradiation to induce rapid tumor cell death. At the tissue level, the Trojan bacteria–based drug delivery system was developed for GBM photothermal immunotherapy by exploiting bacteria-mediated tumor homing. In this system, bacteria loaded with glucose polymer and photosensitive indocyanine green (ICG)-silicon nanoparticles preferentially accumulate in tumor tissue. Upon 808 nm laser irradiation, ICG generates a strong photothermal effect that destroys both the carrier bacteria and adjacent tumor cells. The resulting bacterial debris and released tumor antigens further stimulate antitumor immune responses, prolonging survival in GBM-bearing mice, while residual bacteria are efficiently cleared [120] (Fig. 4A). This strategy exemplifies the most basic photothermal paradigm, in which localized thermal ablation of residual lesions is achieved together with limited but spatially confined immune activation. Moving from tissue-level ablation to organelle-specific targeting, copper sulfide nanoparticles functionalized with arginine-glycine-aspartic (RGD) and trans-activator of transcription (TAT) peptides (CuS@MSN-TAT-RGD NPs) were engineered to achieve nuclear targeting, enabling direct heat generation within the nucleus under 980 nm NIR irradiation. This nuclear-localized hyperthermia rapidly elevates intranuclear temperature, causing irreversible genetic material destruction and inducing exhaustive apoptosis in residual cancer cells. In vivo, a single 5-min laser treatment was sufficient to eliminate tumor cells and prevent recurrence, demonstrating markedly enhanced clearance efficiency compared with cytoplasmic photothermal damage [121]. This approach shifts thermal injury from the tissue or cellular level toward direct genomic disruption, further lowering the threshold for effective eradication of residual tumor cells. Related work further suggests that improving thermal precision may become an important direction in future PTT design for residual tumor settings, where reducing overheating-associated collateral injury and inflammation is particularly desirable, and sequential 808 and 1064 nm irradiation offers one possible strategy to separate sublethal priming from subsequent ablation [122].

Beyond increasing direct cytotoxicity, PTT can also trigger immune pathways, bridging local ablation and systemic immunity, as reflected by multifunctional nanoplatforms that integrate photothermally assisted therapy with ICD-associated immune activation and broader immune microenvironment reprogramming [123]. Prussian blue–based photothermal nanosystems (PBM) was composed of Food and Drug Administration (FDA)-approved PB NPs doped with Mn (III). PTA induces cytosolic DNA release that activates the cGAS-STING pathway, while PBM further amplifies this response by consuming intracellular glutathione and releasing Mn(II), boosting innate immune signaling and promoting antitumor immunity against both local and distant tumors in CT26 colon and 4T1 breast cancer models [124] (Fig. 4B). Building on immune activation, the Fe-doped polydiaminopyridine (Fe-PDAP)@ICG@POM-1 (FIP) nanoplatform integrates PTT with immunometabolic regulation. Upon NIR irradiation, FIP induces ICD while the CD39 inhibitor POM-1 blocks ATP-to-adenosine conversion, alleviating immunosuppressive signaling. Combined with PD-1 ICB, this dual-directional strategy suppresses primary and metastatic tumors and establishes long-term immunological memory [125].

The most advanced photothermal strategies combine local ablation, immune modulation, and microenvironment regulation to maximize residual tumor clearance. JQ-1@PSNs-R silica-based core-shell nanoparticles combine polydopamine-enhanced photothermal effects with delivery of the BRD4 inhibitor JQ-1, where surface roughening improves cellular internalization and drug delivery, leading to effective melanoma eradication, tumor-specific immune activation, and prevention of metastasis and recurrence [126]. Complementary vascular and stromal modulation is exemplified by VI@Gd-NPs, which integrate the vascular-disrupting agent Vadimezan with ICG-mediated PTT and MRI guidance, improving intratumoral ICG delivery, inducing comprehensive ablation, and functioning as an in situ tumor vaccine with CD8^+^ T cell-dependent immunity [127].

#### 4.1.2 Photodynamic therapy

Recent studies have expanded the design framework of photoresponsive nanomedicine by demonstrating precise biological targeting with NIR-II phototheranostics [128], targeted delivery integrated with diagnostic functions for precision treatment [129], and molecularly intelligent photosensitizers activated by disease-associated cues such as pH, redox imbalance, and enzymatic activity [130]. The translational scope of these concepts is further illustrated by sunlight-activated PDT nanoplatforms in diabetic wound infection [131]. In oncology, such design principles are further exemplified by a pH-responsive charge-reversal liposomal system that integrates chemotherapy with synergistic PTT/PDT, while promoting ICD and attenuating therapy-associated PD-L1 upregulation [132]. In residual tumor settings, PDT-based nanoplatforms eliminate residual tumors primarily through light-triggered ROS generation, providing a non-thermal cytotoxic modality well suited for postoperative margins. In implantable postoperative settings, an in situ vaccine hydrogel (APHP-CCCA) has been developed for oral squamous cell carcinoma (OSCC), integrating δ-ALA-mediated PDT, mild PTT, and local immunomodulation by anti-CD47 and CaCO_3_. After 660/808 nm irradiation, it induces tumor cell death and ICD, marked by calreticulin (CRT) exposure and high mobility group box 1 (HMGB1) translocation, while promoting M1 macrophage polarization, DC maturation, increased CD8^+^ and CD4^+^ T cell infiltration, and reduced Treg accumulation. Notably, the immune response was sustained, as effector and central memory T cell populations remained elevated at day 30 and resistance to tumor rechallenge was still evident at day 60 [133] (Fig. 4C). Beyond implantable systems, carrier-minimized phototherapeutic platforms may also contribute to the elimination of residual tumor cells that escape initial phototherapy. A self-delivery chimeric peptide (CCP) combines low-temperature PTT with PDT through tumor cell membrane targeting and induces CRT exposure, HMGB1 release, ATP depletion, and HSP70 upregulation after irradiation. In vivo, CCP increased DC maturation and CD8^+^ T cell infiltration, and experimental depletion of CD8^+^ T cells led to tumor recurrence in two mice by day 13, indicating that CD8^+^ T cells contributed to sustained tumor control. CCP also suppressed tumor growth, reduced experimental lung metastasis, and improved survival over 60 days [134]. Likewise, multifunctional hydrogels expand PDT toward metabolic intervention, as exemplified by the Ru/GOx@Hydrogel, in which ruthenium nanorods provide photodynamic and photothermal effects while glucose oxidase induces starvation therapy. Under irradiation, these mechanisms synergistically eradicate residual melanoma cells and concurrently promote wound healing, highlighting the adaptability of PDT-centered platforms in postoperative settings [135]. At a more integrated level, PDT is incorporated into theranostic nanoplatforms that span diagnosis, surgery, and postoperative treatment. An AIEgen-based system, DDTB, enables NIR fluorescence imaging for preoperative tumor visualization and image-guided resection, followed by intraoperative PTT/PDT of residual lesions. In tumor-bearing mice, this strategy reduced the recurrence rate to 10% by day 25 and prolonged median overall survival to >70 days. When combined with PD-L1 blockade, this platform further enhanced tumor elimination and antitumor immune responses, as reflected by increased CD4^+^ and CD8^+^ T cell infiltration and elevated tumor necrosis factor-α (TNF-α) and interferon-γ (IFN-γ) [136]. Together, these PDT-based strategies illustrate a progression from localized oxidative ablation toward multimodal, immune-enabled perioperative tumor control.

#### 4.1.3 Sonodynamic therapy

SDT-based nanoplatforms utilize ultrasound to activate sonosensitizers, generating ROS in deep tissues without relying on light or heat, effectively eliminating postoperative residual tumors in anatomically shielded regions [137]. Porous mesoporous silica-based sonodynamic nanoparticle (PMSN) has been developed to enhance ultrasound-triggered ROS production within residual tumor tissues. Upon sonication, PMSN induces pronounced oxidative damage and pyroptosis-associated tumor cell death, accompanied by increased TNF-α, IFN-γ, and IL-6 levels and broader immune-cell infiltration, including CD4^+^ and CD8^+^ T cells, B cells, M1-like TAMs, NK cells, and DCs, in both treated and distant tumors. When combined with PD-L1 ICB, this SDT-mediated immune activation suppresses both residual primary tumors and distant lesions by Day 31, demonstrating that ultrasound-triggered oxidative therapy can be effectively integrated with systemic immunotherapy for postoperative tumor control [138] (Fig. 4D).

#### 4.1.4 Radiotherapy or radiofrequency ablation

Nanomaterial-assisted radiotherapy and RFA strategies aim to enhance local energy deposition and overcome residual tumor resistance by integrating nanoplatforms with radiosensitization, catalytic amplification, or programmed cell death induction, which potentiate regulated cell death and systemic immune responses. In radiotherapy, copper-based polyoxometalate nanocapsules (PWCu nanocapsules) sensitize residual tumors to radiotherapy and induce cuproptosis. Upon irradiation, these nanocapsules enhance radiation-induced damage and disrupt copper homeostasis, thereby triggering cuproptosis and overcoming radioresistance. This process is also accompanied by HSP70 upregulation, CRT exposure, HMGB1 release, and CD8^+^ T cell-dependent antitumor immunity, illustrating that nanomaterials can extend radiotherapy beyond direct cytotoxicity [139]. Platforms centered on RFA have increasingly incorporated ferroptosis or ICD to potentiate antitumor efficacy. For instance, Fe-Tirapazamine (TPZ) NPs combine RFA-induced thermal stress with Fenton reaction-mediated ROS generation and hypoxia-activated TPZ cytotoxicity, thereby promoting ICD in residual tumors, as indicated by ATP release, CRT exposure, and HMGB1 secretion. This was accompanied by M1 macrophage polarization, increased CD4^+^ and CD8^+^ T cell infiltration, reduced Treg accumulation, and enhanced Th1 responses, supporting improved residual tumor control and post-ablation immune remodeling [140]. Building on this concept, the hemin and LOX co-loaded CaCO_3_-encapsulated PLGA (HLCaP) nanoreactors combine RFA with ferroptosis induction and ICB to improve control of residual tumors. RFA-mediated tumor disruption enhances nanoparticle retention and penetration, while iron-catalyzed lipid peroxidation promotes ferroptotic death together with HMGB1 release and CRT exposure. In combination with anti-PD-1, this treatment further promotes DC maturation, increases intratumoral CD8^+^ and CD4^+^ T cell infiltration, elevates TNF-α and IFN-γ, and thereby suppresses both distant tumors and postoperative recurrence [141]. Notably, a PEG-modified Fe-based single-atom nanozyme (P@Fe SAZ) enabled low-temperature radiofrequency dynamic therapy to target residual HCC after incomplete ablation. Under radiofrequency stimulation, the nanozyme generated ROS that eradicated residual tumor cells while reversing the post-ablation immunosuppressive microenvironment by promoting macrophage polarization toward an antitumor phenotype, reducing suppressive myeloid cells and Tregs, and enhancing DC maturation and CD4^+^ and CD8^+^ T cell responses [142]. Collectively, these examples illustrate the evolution from local radiotherapy or RFA enhancement toward multifunctional nanoplatforms capable of coordinating regulated cell death with durable immune surveillance.

Overall, these physical and phototherapeutic modalities differ not only in how they eliminate residual tumor cells, but also in the form, magnitude, and durability of immune engagement they trigger. PDT is more consistently associated with canonical ICD-related danger signaling, leading to efficient antigen release, DC activation, and subsequent T cell priming. In contrast, SDT is more frequently linked to pyroptosis-associated inflammatory responses, which can promote broader innate and adaptive immune recruitment but may also introduce variability in immune polarization. Radiotherapy- or RFA-based platforms more often involve noncanonical regulated cell death together with pronounced post-ablation microenvironment remodeling, including vascular disruption and stromal reorganization, which can both facilitate and constrain immune activation. These differences suggest that each modality may engage distinct immune pathways with varying dependence on combination strategies such as ICB. Durable systemic immune memory, however, has been demonstrated only in selected platforms, highlighting the need for more consistent translation of local immune activation into long-term protection.

### 4.2 Chemistry- and metabolism-driven synergistic therapy

Chemistry- and metabolism-driven strategies exploit intrinsic vulnerabilities of tumor cells, combining targeted metabolic disruption with chemical reaction-mediated cytotoxicity to eliminate residual tumors after surgery. Mitochondria-targeted nanoplatforms provide a clear demonstration of this principle. A Prussian blue–based nanoplatform co-doped with calcium and copper and functionalized with a Cur-derived mitochondrial ligand delivers Ca^2+^ and Cu^2+^ directly to melanoma cell mitochondria, disrupting ion homeostasis and amplifying cuproptosis. Under mild NIR-II photothermal activation, it achieves robust suppression of residual tumor growth while simultaneously enhancing angiogenesis and collagen deposition at surgical sites, accelerating wound healing alongside tumor eradication [143] (Fig. 4E). Extending this concept, a hyaluronic acid-based polymer prodrug nanoplatform (CHH-T/NPs) integrates α-cyano-hydroxycinnamic acid, hydroxychloroquine, and mitochondria-targeting IR820 to impair mitochondrial function, inhibit lactate transport, and block protective autophagy in residual colorectal cancer (CRC) cells. This coordinated metabolic and autophagy blockade effectively suppresses cancer stemness, markedly reducing chemoresistance and postoperative recurrence [144]. Together, these examples illustrate how precise subcellular targeting and multi-pathway metabolic interference can be harnessed to maximize cytotoxic effects against residual tumor cells.

Chemical and metabolic strategies can also be extended to generate reactive species in situ and to reshape the TME, further enhancing residual tumor clearance. An injectable GOx@MnCaP@fibrin hydrogel integrates glucose depletion with Fenton reaction–mediated hydroxyl radical generation, selectively killing residual IDH1(R132H) glioma cells, demonstrating the synergy of metabolic starvation and chemical cytotoxicity. This hydrogel system also leverages the slow-release fibrin matrix to maintain sustained ROS production, enhancing the long-term eradication of residual tumor cells while minimizing damage to surrounding healthy tissue [145]. Beyond direct tumor killing, intratumoral metabolic reprogramming can facilitate immune-mediated eradication. Lactate oxidase (LOX)-MnO_2_ @Gel, an injectable lactate oxidase–loaded mesoporous MnO_2_ hydrogel, catalytically reduces intratumoral lactate in HCC after thermal ablation, transforming the immunosuppressive microenvironment into an immunocompetent niche. By depleting lactate, LOX-MnO_2_ @Gel not only inhibits MDSC accumulation but also promotes DC activation and cytotoxic T lymphocyte (CTL) infiltration, effectively turning the tumor site into an in situ immune-activating platform. In combination with ICB therapy, this metabolic remodeling significantly inhibits residual tumor regrowth and lung metastasis while prolonging survival [146].

### 4.3 Temporally programmed therapeutic systems

Residual tumors after surgery are often heterogeneous and resistant to single-modality therapy, motivating the development of temporally programmed, multi-mechanistic treatment strategies. To address this challenge, a trimodal therapy system was designed by integrating PTT, chemodynamic therapy (CDT), and dual-drug chemotherapy into a single nanoparticle platform. The nanoparticles consist of Fe-coordinated polydopamine cores, copper peroxide nanocores, and surface-decorated DOX–Fe(III)–gossypol infinite coordination polymers (ICPs), enabling sequential activation of each modality. Upon tumor accumulation, NIR irradiation triggers PTT, causing rapid thermal ablation of tumor cells. The copper peroxide cores then generate ROS via Fenton-like reactions, inducing chemodynamic cell death in residual tumor cells, followed by controlled release of DOX and gossypol to target proliferative and drug-resistant populations. In PC-3 xenograft models, this programmed approach achieved nearly complete tumor inhibition with no recurrence for 60 days and minimal systemic toxicity [147]. Similarly, a Janus hydrogel platform (GA@CaMP) integrating sonosensitive MHP nanocomposites and oxygen-releasing CaO_2_ NPs enabled spatiotemporally controlled therapy of residual bone metastatic lesions, in which elevated ROS under ultrasound stimulation cooperated with ZBP1 upregulation to trigger necroptosis and eliminate tumor cells, whereas lower ROS levels subsequently promoted osteogenic gene expression and bone regeneration; sustained oxygen release further alleviated hypoxia, enhancing both antitumor efficacy and tissue repair [148] (Table 3, Supplementary Table 3).

**Table 3:** Nanomaterial-enabled synergistic mechanisms for residual tumor treatment.

Nanoplatform	Synergistic modality	Mechanism	Tumor model	Synergistic efficacy	Ref.
PBM	PTT	Activating cGAS–STING signaling via cytosolic DNA release and Mn(II)-mediated amplification	Residual colon cancer and breast cancer after iPTA	Inducing systemic antitumor immunity against local residual and distant tumors	[124]
APHP-CCCA	PDT + PTT + calcium overload therapy	Inducing apoptosis of residual OSCC cells, promoting ICD and M1 macrophage polarization, activating DCs and CTLs	Postsurgical residual OSCC	81.86% tumor regression, inhibiting pulmonary metastasis, establishing long-term antitumor immunity	[133]
PMSN	SDT	Inducing ROS-mediated pyroptosis, amplifying tumor oxidative stress	Liver cancer	85.93% treated and 77.09% distant tumor suppression, ↑PD-L1 blockade efficacy	[138]
HLCaP nanoreactors	RFA	Driving iron-catalyzed lipid peroxidation and activating systemic immunity	Residual liver and breast cancer after iRFA	4/5 recurrence-free at 90 days, ↓metastasis, ↑ICB efficacy, ↑median survival to 72 d	[141]
CHH-T/NPs	Metabolic inhibition + PTT	Inhibiting lactate transport, mitochondrial function, and protective autophagy	Postoperative subcutaneous residual CRC	Suppressing chemoresistance, preventing tumor recurrence, prolonging survival	[144]
LOX-MnO_2_@Gel	Lactate metabolism modulation	Depleting intratumoral lactate, remodeling immunosuppressive TME, enhancing ICB efficacy	Residual HCC after iMWA	Inhibiting recurrence, ↓20% lung metastasis, ↑median survival to 47.5 d	[146]
ICPs@PDA:CuO_2_ NPs	PTT + CDT + dual chemotherapy	Activating thermal ablation, ROS generation and controlled drug release to clear tumor cells	Subcutaneous prostate cancer	Achieving nearly 100% tumor inhibition and preventing recurrence within 60 days	[147]

## 5 Nanomaterial-driven immune modulation for residual tumor control and antitumor effects

Although nanomaterial-based strategies have substantially improved the local management of residual tumors through intraoperative clearance, localized postoperative drug delivery, and multimodal therapeutic reinforcement, durable postoperative benefit ultimately depends on whether these local interventions can be translated into sustained antitumor immunity. In this context, nanotherapy-induced ICD represents a central initiating event within a broader immunological cascade. By inducing the release of tumor-associated antigens together with danger-associated molecular patterns, ICD promotes the recruitment and activation of antigen-presenting cells, particularly DCs, thereby facilitating antigen processing, presentation, and subsequent T cell priming within the residual tumor microenvironment. As these responses are amplified and local immunosuppressive barriers are alleviated, activated cytotoxic lymphocytes may exert antitumor effects beyond the treated residual niche, extending therapeutic impact to distant sites. In combination with ICB or other immunoregulatory interventions, this process may further enhance the expansion and persistence of tumor-specific T cell responses, supporting the development of long-term immune memory and more durable systemic protection against recurrence and metastasis.

### 5.1 Nanotherapy-induced immunogenic cell death in residual tumors

Related studies in solid tumor models have shown that nanoplatforms can link local tumor killing to downstream immune activation, including ICD induction, cGAS-STING signaling, and enhanced dendritic-cell and CD8^+^ T cell responses [149], thereby providing a mechanistic basis for similar strategies in residual tumor settings. In residual tumors, this principle is especially relevant, as ICD can help translate local cytotoxic interventions into broader systemic antitumor immunity. Accordingly, nanotherapeutic platforms developed for post-ablation or postoperative settings often use ICD as a central immunological trigger to amplify danger-signal release, enhance antigen presentation, and remodel the local immune microenvironment. This allows residual lesions to function as immunologically active niches rather than merely passive targets of cytotoxic therapy [150,151].

To address residual disease following incomplete local ablation, nanocarriers that impose metabolic stress and amplify autophagy have been shown to reinforce ICD at the ablation margin. ZIF-8 NPs loaded with the autophagy activator STF62247 (STF62247@ZIF-8/PEG-FA NPs) were developed for therapy after iRFA. These nanoparticles markedly enhanced autophagy in residual tumor cells, induced robust ICD, and promoted DC maturation, thereby suppressing tumor regrowth and supporting immunological memory. When combined with the PD-L1 inhibitor BMS202 (STF62247-BMS202@ZIF-8/PEG-FA NPs), the platform further intensified ICD induction while alleviating local immunosuppression, resulting in significantly enhanced antitumor efficacy [152].

For the distinct challenge of preventing recurrence after surgical resection, injectable in situ–forming hydrogels have emerged as effective platforms for sustained and spatially confined ICD induction within postoperative cavities. An injectable zwitterionic hydrogel (P-DOX/1 MT@MM-Gel), incorporating DOX and the IDO pathway inhibitor 1-methyl-tryptophan (1-MT) on a dendrimer core and coated with macrophage membranes, enhanced tumor cell recognition and uptake. This system induced ICD, promoted DC maturation, increased cytotoxic T cell infiltration, and substantially reduced postoperative recurrence through sequential drug release and microenvironmental reprogramming [153]. A complementary approach employed a PSBMA-based injectable hydrogel delivering macrophage membrane–coated CuO_2_/DOX NPs together with the STING agonist 2′,3′-cGAMP (Gel@M/CuO_2_/DOX/STING). In this system, STING activation directly stimulated innate immune signaling, while M/CuO_2_/DOX induced DNA damage, ICD, and copper-mediated tumor cell death. Together, these effects enhanced DC maturation and CD8^+^ T cell infiltration, thereby suppressing tumor recurrence and metastasis [154].

**Fig. 5:**
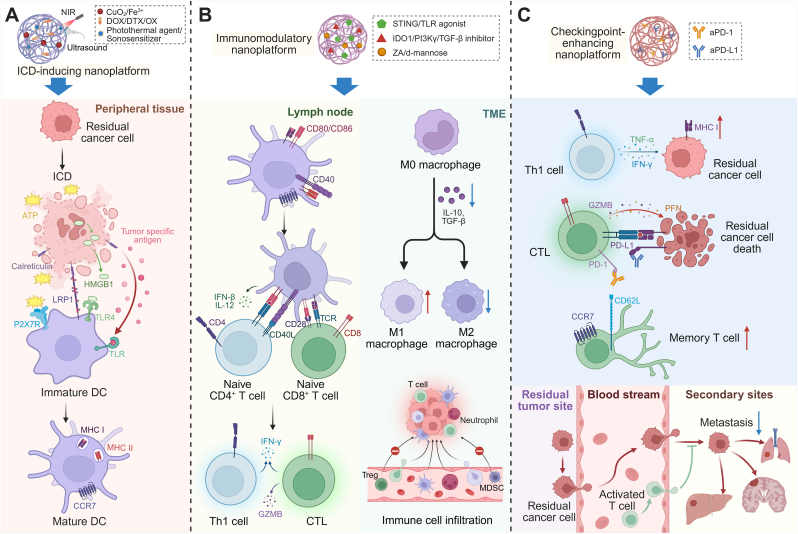
**Nanomaterial-driven immune modulation for residual tumor control and durable antitumor immunity.** (A) An ICD-inducing nanoplatform accumulates in peripheral residual tumor tissue after surgery or ablation. Upon activation by NIR irradiation or ultrasound, the platform triggers ICD in residual cancer cells, accompanied by CRT exposure, ATP release, and HMGB1 secretion. These damage-associated molecular patterns (DAMPs) promote DC recruitment and maturation. (B) Nanomaterial-mediated immune activation reshapes the residual TME by alleviating myeloid-driven immunosuppression, including macrophage reprogramming and inhibition of MDSC and Treg accumulation, and activating innate immune signaling pathways such as STING. These effects enhance immune cell infiltration, DC activation, and effective priming of tumor-specific CD4^+^ and CD8^+^ T cells. (C) Nanoplatform-assisted delivery of immune checkpoint inhibitors targeting the PD-1/PD-L1 axis reinvigorates exhausted CTLs in residual tumors. In coordination with prior immune priming, checkpoint blockade sustains effector T cell function and promotes memory T cell formation, enabling durable immune surveillance against tumor recurrence and metastasis.

Beyond sustained local delivery, nanoplatforms integrating chemical, photothermal, and immunological mechanisms further strengthened ICD induction. Docetaxel (DTX)-loaded Zein/CSP-GTP/Fe^III^ NPs functioned as a multifunctional chemo–immuno–photothermal system with pH-responsive drug release and efficient tumor uptake, inducing pronounced ICD, promoting DC maturation, and enhancing T cell infiltration, ultimately eradicating primary tumors and preventing relapse and metastasis [155]. Similarly, a gelatin–metal–polyphenol hydrogel (Gel-Fe-PA), capable of in situ generation of Fe-PA NPs, produced localized photothermal heating under NIR irradiation, eliminated postoperative residual tumors, and induced canonical ICD hallmarks, including ATP, CRT, and HMGB1 release, thereby activating DCs and eliciting systemic antitumor immunity [156] (Fig. 5A).

### 5.2 Nanomaterial-mediated immune activation within the residual tumor microenvironment

Beyond direct tumor cytotoxicity, nanomaterial-based platforms have been increasingly leveraged to activate antitumor immunity by alleviating local immunosuppression, enhancing immune cell recruitment, and coupling cytotoxic therapies with immune amplification. A recently reported Fe_3_O_4_ NPs@Met-GA hydrogel also exemplified this immunomodulatory design by integrating ICD induction with macrophage reprogramming, PD-L1 downregulation, and enhanced cytotoxic T cell infiltration within a single platform [157]. Particularly in postoperative or post-ablation settings, these strategies aim to convert residual tumor sites into immunologically active niches capable of supporting durable systemic immune responses.

A first class of nanomaterials focuses on relieving myeloid-driven immunosuppression following local thermal ablation, thereby restoring immune responsiveness in residual tumors. A biohydrogel scaffold (OX&IPI549@Gel) was developed for localized chemoimmunotherapy after incomplete MWA (iMWA), enabling the co-delivery of oxaliplatin (OX) and the PI3Kγ inhibitor IPI549. By simultaneously inducing tumor cell damage and targeting immunosuppressive myeloid cells, this system suppressed tumor growth and metastasis while establishing long-term immune memory against tumor rechallenge [158]. In a complementary approach, the Bi-MOF-l-Cys@PEG@HA (BMCPH) nano-immunomodulator was designed to enhance microwave thermal therapy and reverse immunosuppression in residual tumors. Upon microwave irradiation, BMCPH released l-cysteine to generate hydrogen sulfide (H_2_S) in situ, which inhibited MDSC accumulation, scavenged ROS, and promoted CTL infiltration. Hyaluronic acid functionalization further facilitated immune cell recruitment, collectively reducing tumor recurrence and metastasis after thermal ablation [159].

Targeting immunosuppressive immune cell populations within the residual tumor milieu represents another effective strategy to amplify immune activation. d-mannose–chelated iron oxide nanoparticles (man-IONPs) were engineered to selectively accumulate in peri-ablation zones following iMWA and to reprogram M2-like macrophages toward an antitumor M1 phenotype in HCC. This macrophage polarization alleviated local immune suppression and significantly inhibited tumor progression in vitro and in vivo [160]. Similarly, the ICG-SB@Lip-ZA nanosystem integrated PTT with immune modulation by combining ICG, zoledronic acid (ZA), and the TGF-β inhibitor SB-505124. In this system, PTA induced tumor cell death, ZA depleted M2-like TAMs, and SB-505124 suppressed CAF expansion, collectively reshaping the immune landscape, enhancing T cell infiltration, and achieving robust tumor eradication in breast cancer models [161].

Beyond modulation of the local immune milieu, postoperative immunotherapy platforms have been developed to directly activate both innate and adaptive immune pathways. A polymeric hydrogel loaded with decitabine, CDDP, and manganese ions served as a multifunctional immunogel that induced DAC burst gasdermin E (GSDME) -mediated pyroptosis through sustained drug release while activating the STING pathway via Mn^2+^. This approach elicited potent antitumor immune responses, resulting in prolonged survival in both recurrent and metastatic tumor models [162]. In a related strategy, nanoexosome-loaded porous microneedles (MNs) were designed for precise intratumoral delivery of the STING agonist MSA-2. Upon ultrahigh dose-rate FLASH irradiation, exosome-mediated release of MSA-2 activated STING signaling, elevated type I interferon production, and promoted DC maturation, thereby suppressing recurrence with minimal systemic toxicity [163].

Importantly, injectable hydrogels and self-assembling peptide systems have been exploited to establish sustained immunoactive niches and reinforce ICD. An injectable hydrogel incorporating a tumor-homing immune nanoregulator was developed for post-resection treatment of GBM multiforme, where it induced ICD, inhibited indoleamine 2,3-dioxygenase-1 (IDO1), and promoted T cell infiltration through sustained release of CXCL10, effectively suppressing tumor recurrence [164]. In parallel, the AIEgen-conjugated self-assembling peptide TPA-FFG-LA formed nanoassemblies on epidermal growth factor receptor (EGFR)-expressing TNBC cells, where it inhibited EGFR signaling, induced lysosomal membrane permeabilization, and generated ROS upon light irradiation. This cascade triggered robust ICD and immune activation, leading to effective tumor suppression in vitro and in vivo [165] (Fig. 5B).

### 5.3 Immune checkpoint blockade–based modulation and long-term immune memory in residual tumors

Effective control of residual tumors and durable prevention of recurrence require not only the elimination of therapy-resistant tumor cells but also the reinvigoration of exhausted T cells and the establishment of long-term immune memory. ICB, particularly targeting the PD-1/PD-L1 axis, has emerged as a central strategy to restore cytotoxic T cell function [166]. However, current evidence indicates that ICB monotherapy has important limitations in residual tumor settings, with clinical benefit often restricted to a subset of patients and durable control remaining difficult to achieve. In post-ablation models, iRFA can accelerate residual tumor progression and establish an immunosuppressive microenvironment that compromises PD-1 blockade efficacy [30]. PD-1/PD-L1 blockade after RFA likewise produced only transient tumor control, and the accompanying retrospective case-controlled clinical analysis did not show an obvious prolongation of progression-free survival with the addition of PD-1 blockade after RFA [167]. Postoperative niches may also remain immunosuppressive, further limiting the efficacy of ICB [168]. Consistent with this, in a phase III clinical trial involving patients with residual pathological disease after neoadjuvant chemoradiotherapy, adjuvant nivolumab significantly prolonged disease-free survival, but recurrence remained common; median disease-free survival was 22.4 months versus 11.0 months in the placebo group, while grade 3-4 treatment-related adverse events occurred in 13% of patients and 9% discontinued treatment because of toxicity [169]. These findings support the clinical rationale for combination strategies, particularly those integrating nanomaterial-mediated local interventions to induce ICD or remodel the residual tumor microenvironment, thereby improving the depth and durability of antitumor immunity.

Photothermal- and energy-based nanotherapies provide an effective means to initiate antitumor immune priming by inducing ICD and antigen release at residual tumor sites. In basal-like breast cancer, erythrocyte membrane-coated black phosphorus quantum dots (BPQD-RMNV) enabled efficient PTA under NIR irradiation, leading to tumor cell apoptosis, antigen liberation, and DC activation. Concurrent PD-1 blockade prevented CD8^+^ T cell exhaustion, resulting in robust suppression of residual and metastatic lesions and enhanced systemic cytotoxic T cell responses [170]. Similarly, platelet-mimicking nanocarriers (P-P-IO) co-delivering iron oxide nanoparticles and anti-PD-L1 antibodies (aPD-L1) preferentially accumulated at postsurgical sites. Local PTT induced necrosis of residual tumor cells and antigen release, while simultaneous PD-L1 blockade amplified T cell infiltration and activation, thereby reducing postoperative recurrence [171].

Beyond photothermal modalities, nanoplatforms have been designed to potentiate immunogenicity by alleviating hypoxia and enhancing oxidative stress. An oxygen self-enriching nanodrug (PCL@O_2_), incorporating a sonosensitizer and an oxygen reservoir, markedly enhanced SDT following RFA. By promoting ROS generation, ICD induction, DC maturation, and CTL activation, this system synergized with PD-1 blockade to suppress tumor progression, reduce regulatory T cell infiltration, and establish durable immune memory [172]. In a complementary approach, the radio-immunostimulant IPI549@HMP catalyzed endogenous H_2_O_2_ to generate oxygen, relieving hypoxia and sensitizing tumors to radiotherapy. This microenvironmental reprogramming significantly enhanced responsiveness to anti-PD-L1 therapy, resulting in effective control of both residual and distant tumors and long-term protection against tumor rechallenge [173]. Multifunctional nanodevices further integrate cytotoxic therapy, vascular regulation, and immune checkpoint inhibition to reinforce antitumor immunity. Portable nanofiber patches composed of germanium phosphorus and anlotinib (AL) provided trimodal therapy for postoperative HCC. NIR irradiation triggered drug release, inhibited angiogenic signaling, and induced ICD. When combined with PD-L1 blockade, this strategy enhanced T cell infiltration, suppressed residual tumor growth and metastasis, and reinforced immune-mediated tumor control [174] (Fig. 5C) (Table 4, Supplementary Table 4)

**Table 4:** Nanomaterial-driven immunomodulatory strategies for residual tumor control.

Nanomaterial	Immunomodulator	Immune effect	Tumor model	Immune outcome	Ref.
STF62247@ZIF-8/PEG-FA (SZP) or STF62247-BMS202@ZIF-8/PEG-FA (SBZP) NPs	STF62247; BMS202	Inducing autophagy-amplified ICD and enhancing antigen presentation	Subcutaneous residual HCC after iRFA	Establishing immune memory, 71.00% (SZP)/86.76% (SBZP) tumor suppression	[152]
Gel@M/CuO_2_/DOX/STING	2′,3′-cGAMP	Activating STING signaling; inducing ICD, promoting DC maturation and enhancing CD8^+^ T cell infiltration	Postoperative subcutaneous breast cancer	100% tumor suppression, metastasis-free via enhancing antitumor immunity	[154]
OX&IPI549@Gel	IPI549	Relieving myeloid-driven immunosuppression, inducing ICD, promoting DC activation, enhancing T cell infiltration	Residual subcutaneous CRC after iMWA	33.3% tumor suppression, metastasis-free at day 17, eliciting long-term antitumor immunity	[158]
Decitabine/CDDP/Mn^2+^ polymeric hydrogel	Decitabine, CDDP, Mn^2+^	Inducing pyroptosis; activating STING-mediated innate immunity; promoting adaptive immunity activation	Postoperative residual subcutaneous breast tumor	↑antitumor immunity; 40% cure and 80% survival at day 36 for recurrence; 60% survival at day 100 for metastasis	[162]
BPQD-RMNV	BPQDs	Inducing photothermal ICD, recruiting DCs and activating CD8^+^ T cells	Residual TNBC after PTT	Suppressing residual and metastatic tumor growth, enhancing ICB efficacy	[170]
PCL@O_2_	Ce6 (ICD inducer)	Enhancing ROS-mediated ICD, promoting DC maturation, activating CTLs	Residual tumor after iRFA	77.13% primary and 72.41% distant tumor suppression	[172]

Despite these encouraging results, durable control is not consistently achieved across residual tumor models, indicating that postoperative immunotherapy remains constrained by adaptive resistance, response heterogeneity, and limited efficacy of single-agent strategies. In several postoperative settings, monotherapy produced only partial tumor control. For example, free chimeric antigen receptor T (CAR-T) cells, with or without anti-PD-L1, showed only moderate antitumor activity and were associated with marked PD-L1 upregulation in tumor cells, suggesting the emergence of adaptive immune resistance during therapy [175]. Similarly, in resected B16F10 tumors, either an anti-PD-L1-loaded hydrogel (Gel@aPD-L1) or a hydrogel loaded with cRGD-modified redox-responsive Withaferin A prodrugs (Gel@WA-cRGD) alone showed limited control and failed to prevent local recurrence, whereas the combined Gel@WA-cRGD + anti-PD-L1 hydrogel achieved superior efficacy [176]. In residual HCC after iRFA, a TAK-981@bovine serum albumin nanoparticle-loaded poly(D,L-lactide)-poly(ethylene glycol)-poly(D,L-lactide) hydrogel (BT-NPs@PLEL) enhanced local immune activation but also induced PD-L1 upregulation, while both BT-NPs@PLEL and anti-PD-L1 monotherapy remained only moderately effective, and even the combination achieved complete eradication in only 1 of 6 tumors [95]. In addition, therapeutic responses were context-dependent. In the intraoperative poly(I:C) hydrogel model, blockade of interferon-α (IFN-α) or the IFN-α/β receptor abolished the antitumor effect, indicating that effective postoperative immune activation may depend on specific host signaling pathways rather than being broadly reproducible across residual tumor settings [177]. Furthermore, postoperative residual tumors may remain difficult to remodel because surgery itself can intensify local immunosuppression, as reflected by increased CXCR4+ tumor cells, Tregs, MDSCs, and PD-L1-positive cancer cells after resection in orthotopic breast cancer [178]. Together, these findings indicate that the clinical relevance of postoperative immunotherapy should be evaluated not only by its capacity to activate antitumor immunity, but also by its susceptibility to adaptive immune escape, its context-dependent variability, and the need to balance immune stimulation with treatment tolerability and safety.

Collectively, these studies suggest that nanomaterial-driven immune modulation in residual tumors can be understood as a unified immunological process, although the strength and durability of such immune protection are not uniformly achieved across all residual tumor settings. ICD induced by local nanotherapies initiates this process through the release of tumor antigens and danger-associated signals, thereby facilitating antigen presentation and triggering immune activation in the residual tumor microenvironment. As these responses are further amplified and local immunosuppression is alleviated, cytotoxic immune cells can exert broader antitumor effects beyond the primary treatment site. In combination with ICB, this process may ultimately promote the formation of long-term immune memory and sustained systemic protection against recurrence and metastasis.

## 6 Multifunctional nanobiomaterials for postoperative therapy

### 6.1 Nanoplatforms combining tumor eradication and tissue repair

Effective management of residual tumors requires not only their complete elimination but also support for tissue regeneration, which is particularly critical for the recovery of bone and soft tissues. Conventional treatments often focus on cytotoxicity, but fail to support functional tissue regeneration. Nanobiomaterials offer a promising approach, combining targeted cytotoxic agents and ECM-mimicking scaffolds to achieve precise tumor clearance while promoting tissue repair [179,180].

**Fig. 6:**
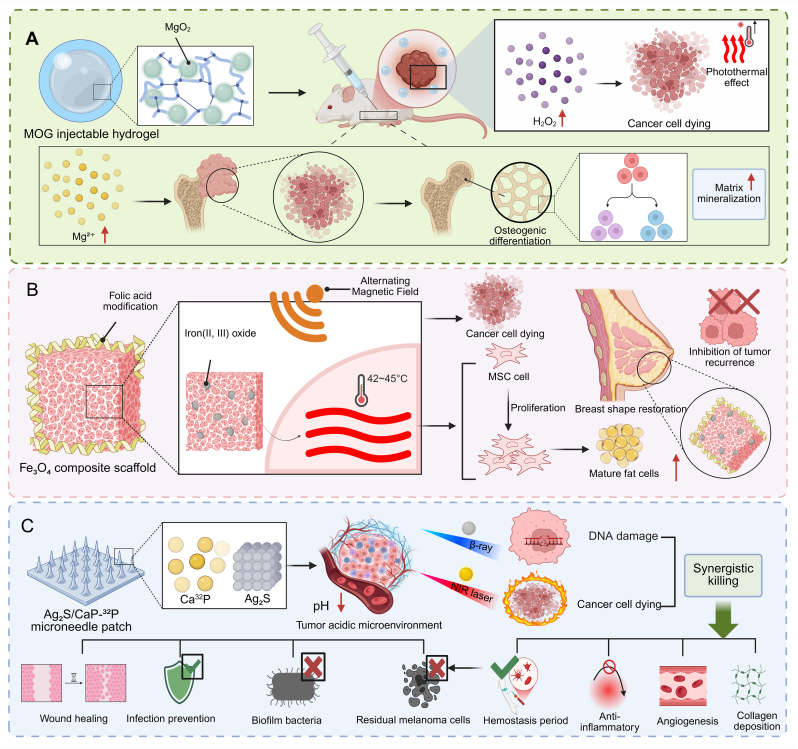
**Multifunctional nanobiomaterials for postoperative tumor treatment and tissue regeneration.** (A) The MOG injectable hydrogel incorporates MgO_2_ NPs that induce photothermal effects under NIR irradiation, enhancing tumor cell death through controlled release of H_2_O_2_, while sustained Mg^2+^ release promotes osteogenesis and bone matrix mineralization. (B) Folic acid-modified Fe_3_O_4_ composite scaffolds, activated by an alternating magnetic field, induce hyperthermia (42-45 °C) to ablate residual tumor cells, promote mesenchymal stem cell proliferation, and aid in the restoration of breast shape by maturing adipose tissue. (C) The Ag_2_S/CaP-^32^P microneedle patch releases β-emitting ^32^P and photothermal Ag_2_S nanodots under acidic tumor conditions, achieving synergistic brachytherapy and PTA of residual melanoma cells and bacteria, while promoting wound healing, angiogenesis, and collagen deposition.

In osteosarcoma, nanoplatforms have been engineered to eradicate residual tumor cells and promote bone regeneration. Chlorogenic acid-gold nanohybrids, self-assembled via Au-catechol interactions, exhibit strong NIR photothermal effects that efficiently induce apoptosis in residual osteosarcoma cells. In addition, mild NIR exposure activates heat shock protein signaling, which enhances osteogenic differentiation and promotes matrix mineralization, contributing to effective bone repair. These nanohybrids also provide the advantage of precise spatial targeting, minimizing damage to surrounding healthy tissue and creating a favorable local microenvironment for bone regeneration [181]. Along the same line, a biomimetic nanofibrous 3D matrix based on bioactive glass nanofibers and genipin-crosslinked gelatin has also been developed for postoperative osteosarcoma, combining photothermal tumor ablation with support for mineralized bone regeneration. This example further highlights the potential of multifunctional scaffolds to integrate residual tumor control, antibacterial activity, and postoperative tissue repair [182]. In another strategy, an injectable in situ cross-linkable hydrogel composed of dopamine-conjugated gelatin and MgO_2_ NPs (MOG) allowed sequential tumor suppression and bone regeneration. Controlled H_2_O_2_ release enhanced PTT to prevent recurrence, while sustained Mg^2+^ release promoted osteogenesis, demonstrating significant antitumor and regenerative efficacy in postoperative osteosarcoma models [183] (Fig. 6A). Further integrated, a 3D-printed porous polylactic acid scaffold filled with GelMA/alginate/borax hydrogel integrates cRGD-modified Cu-Cys-PEG NPs that respond to the acidic TME. This design enables sequential tumor-targeted therapy: the acidic pH triggers glutathione depletion and Fenton-like ROS generation, selectively eliminating residual osteosarcoma cells, while subsequent pH normalization and scaffold degradation provide sustained support for osteogenic differentiation. The scaffold's porous structure facilitates nutrient transport, cell infiltration, and vascularization, further enhancing bone regeneration while maintaining structural integrity post-tumor ablation [184]. Notably, a bionic bilayer scaffold composed of croconaine dye-polyethylene glycol@sodium alginate hydrogel combined with a poly(l-lactide)/hydroxyapatite polymer matrix simultaneously applies PTA and hydroxyapatite-induced mitochondrial dysfunction to trigger tumor cell death, while promoting osteointegration and osteochondral regeneration via upregulation of collagen type-I, osteopontin, and RUNX2. In vivo, the scaffold modulated the osteal microenvironment and facilitated functional osteochondral tissue restoration, demonstrating its potential as a multifunctional platform that integrates tumor eradication with bone and cartilage repair [185].

In breast cancer, multifunctional scaffolds and polymeric systems have been developed to achieve localized tumor eradication while supporting tissue reconstruction. Multifunctional scaffolds incorporating gold nanoparticles provide ECM-like structures and tissue-mimicking mechanical properties, enabling sustained release of AuNPs that actively detect and kill residual tumor cells through folic acid–mediated targeting, surface-enhanced Raman scattering, and photothermal effects. These scaffolds also support cellular adhesion, proliferation, and migration, providing a favorable microenvironment for breast tissue regeneration [186]. Furthermore, folic acid–functionalized Fe_3_O_4_/gelatin composite scaffolds with controlled pore structures exploit alternating magnetic field–mediated hyperthermia to ablate residual tumor cells. The scaffolds enhance mesenchymal stem cell proliferation and adipogenic differentiation, thereby promoting reconstitution of adipose tissue and restoring post-surgical breast morphology. The combination of selective tumor capture, controlled hyperthermia, and tissue-supporting architecture exemplifies how scaffold design can integrate antitumor efficacy with regenerative potential [187] (Fig. 6B). Additionally, DMSN@PCL scaffolds incorporate DOX-loaded mesoporous silica nanoparticles (DMSN) into a polycaprolactone matrix, enabling localized and sustained chemotherapy over nine weeks. In post-surgical mouse models, these scaffolds significantly inhibited local breast tumor recurrence for up to four weeks, demonstrating effective antitumor activity with minimal systemic toxicity. Beyond tumor suppression, the scaffold's biodegradable polycaprolactone framework provides mechanical support for tissue reconstruction and creates a favorable microenvironment for cell adhesion and proliferation, facilitating postoperative breast tissue regeneration [188].

In melanoma and cutaneous tumors, multifunctional local biomaterials, including injectable hydrogels and nanofibrous matrices, provide synergistic antitumor and regenerative effects. A nanocomposite scaffold comprising black phosphorus nanosheets embedded in a gelatin-polycaprolactone nanofibrous matrix was developed for post-melanoma surgery therapy and wound healing. In this system, black phosphorus nanosheets loaded with DOX and conjugated with NH_2_-PEG-FA underwent NIR-triggered sol-gel transition, enabling cooperative PTT and heat-triggered chemotherapy while promoting tissue repair through ERK1/2 and PI3K/AKT activation [189]. Black titania-chitosan (BT-CTS) thermogel, incorporating oxygen-vacancy–rich black titania nanoparticles within a thermosensitive chitosan hydrogel, achieves efficient photothermal and photodynamic ablation of residual cutaneous tumor cells under single NIR irradiation, while the hydrogel matrix supports skin cell adhesion and migration, accelerating wound healing and promoting effective repair of tumor-resection–associated skin wounds [190]. Similarly, a multifunctional nanocomposite hydrogel composed of carboxymethyl chitosan, oxidized fucoidan, and tannic acid-capped gold nanoparticles rapidly kills melanoma cells and bacteria under NIR irradiation, scavenges ROS, and enhances angiogenesis and collagen deposition, thereby preventing tumor recurrence and facilitating skin regeneration. The hydrogel demonstrates good injectability, self-healing, adhesion, and biocompatibility, highlighting its multifunctional potential for postoperative tumor management [191].

### 6.2 Antimicrobial and antitumor nanomaterials

Postoperative infections not only compromise wound healing but also create an immunosuppressive and pro-tumorigenic microenvironment, which can promote residual tumor survival and recurrence [192]. Conventional approaches often fail to control infections while addressing tumor regrowth, highlighting the need for multifunctional materials that integrate anti-infective activity with antitumor therapy. AIE photosensitizers (AIE PSs) illustrate this multifunctional potential. They have been explored for imaging-guided tumor phototherapy with efficient tumor destruction [193], and more recently extended to antibacterial PDT, where pH-responsive ROS amplification enables potent bacterial killing and promotes infected wound healing in acidic microenvironments [194]. This conceptual versatility is further reflected in postoperative nanoplatforms that directly integrate antitumor and anti-infective functions. In superficial lesions such as melanoma, a nanocomposite microneedle patch integrating Ag_2_S/Ca^32^P NPs was developed for melanoma postoperative treatment. Acid-responsive decomposition within tumor and biofilm niches releases β-emitting ^32^P and photothermal Ag_2_S nanodots, achieving synergistic brachytherapy and PTA of residual tumor cells and bacteria. Concurrently, the microneedle matrix promotes scar-free wound healing by reducing inflammation and enhancing angiogenesis, granulation, and collagen deposition. This platform demonstrates how minimally invasive, localized delivery systems can simultaneously eradicate residual tumors and infections while supporting optimal wound recovery [195] (Fig. 6C). Moving to deeper internal tumors, an implantable anti-bacterial and anti-cancer fibrous membrane (AAFM) was developed to prevent pancreatic cancer recurrence. The membrane is prepared by co-electrospinning GEM with poly-L-lactic acid (PLLA), followed by tannic acid–mediated in situ generation of AgNPs. This design provides sustained GEM release, combats Gammaproteobacteria-induced chemotherapy resistance, and effectively inhibits residual cancer cells, enhancing the efficacy of postoperative chemotherapy. The AAFM exemplifies how scaffold-based materials can integrate chemotherapeutic delivery with antimicrobial activity to overcome microbe-mediated drug resistance and reduce tumor relapse risk [196]. For versatile postoperative applications where minimally invasive delivery is desired, a light-activated hydrogel composed of Fe-doped Ag_2_S nanodots (BGN-Fe-Ag_2_S) and poly(ethylene glycol) double acrylates (PEGDA) was developed for post-surgical tumor and infection management. Under laser irradiation, Ag_2_S nanodots induce photothermal gelation, while Fe-doped BGN-Fe-Ag_2_S generates chemodynamic ROS to ablate residual tumor cells and multidrug-resistant bacteria. The hydrogel also accelerates wound healing and skin appendage regeneration, demonstrating its potential as a minimally invasive, multifunctional biomaterial capable of precise tumor eradication, infection control, and tissue repair. This platform highlights the versatility of responsive injectable materials for managing complex postoperative scenarios, particularly where combined antitumor and antimicrobial effects are needed [197].

## 7 Challenges and perspectives

Despite the promise of nanomaterials for postoperative and post-ablation residual tumors, clinical translation remains limited by four major challenges: heterogeneity of residual tumor niches, insufficient mechanistic resolution, limited long-term efficacy and biosafety data, and unresolved barriers in standardization, manufacturing, regulation, and clinical implementation.

**Fig. 7:**
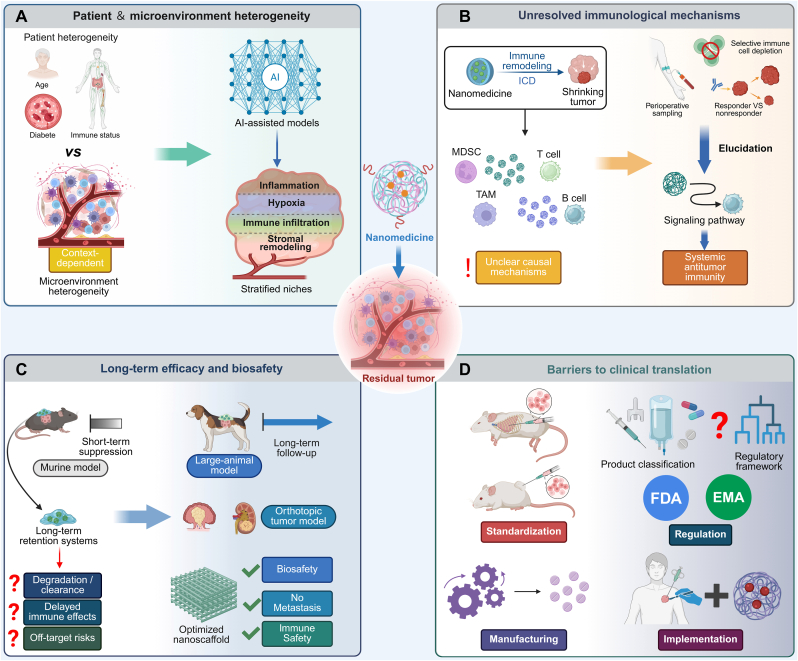
**Challenges and perspectives in nanomaterial-based therapy for postoperative and post-ablation residual tumors.** (A) The pronounced heterogeneity of residual tumors arises from both interpatient variation and context-dependent microenvironmental differences, including inflammation, hypoxia, immune infiltration, and stromal remodeling, highlighting the need for AI-assisted stratification and context-responsive nanomedicine design. (B) Although nanomedicine can promote tumor shrinkage and immune remodeling, the causal relationships linking local immune-cell changes, signaling pathways, and systemic antitumor immunity remain insufficiently resolved, underscoring the importance of perioperative sampling and mechanistic elucidation. (C) Insufficient assessment of long-term efficacy and biosafety remains a major bottleneck, particularly for nanomaterials with prolonged local retention, emphasizing the need for extended follow-up, in vivo fate evaluation, and validation in orthotopic and large-animal models. (D) Clinical translation is further hindered by the lack of standardization in residual tumor models, manufacturing processes, product classification, and regulatory pathways, warranting more clinically relevant evaluation systems and implementation-oriented development frameworks.

First, heterogeneity among patients and postoperative residual tumor microenvironments fundamentally limits the generalizability of nanotherapeutic strategies. Residual tumor biology is shaped not only by tumor type, anatomical location, and treatment modality, but also by patient-specific factors such as age, metabolic status, and immune competence. Aging and frailty can reshape the postoperative niche through immunosenescence, impaired tissue repair, and altered stromal remodeling, thereby influencing both immune responsiveness and therapeutic efficacy [198,199]. Diabetes may further exacerbate these challenges by promoting endothelial dysfunction, chronic inflammation, and altered immune cell infiltration, which can affect drug delivery and local immune activation [200], in some contexts, agents such as metformin have been reported to partially restore antitumor immunity [201]. In addition, immunocompromised conditions, whether disease-related or therapy-induced, may further limit effective immune activation and reduce the durability of treatment responses. Together, these factors highlight the importance of incorporating patient heterogeneity into the design and evaluation of nanomaterial-based strategies for postoperative residual tumors. A more operational framework for postoperative microenvironment stratification may involve categorizing residual tumor niches into inflammation-dominant or immunosuppressive niches, hypoxia-enriched niches, fibrosis- or stroma-dominant niches, immune-excluded or immune-desert niches, and mechanically restrictive niches. These categories can be defined by measurable features related to inflammation, hypoxia, stromal remodeling, immune infiltration, and tissue biomechanics. Cytokine patterns together with myeloid/Treg enrichment indicate inflammation-driven or immunosuppressive niches [202], whereas hypoxia-enriched niches can be identified by hypoxia-related gene-expression or imaging signatures [203,204]. Dense-stroma or fibrosis-dominant niches are characterized by collagen deposition, ECM remodeling, and other fibrosis-associated changes [205]. The balance between CD8^+^ T cell exclusion and inflamed immune infiltration distinguishes immune-excluded from immune-desert niches [206]. while mechanically restrictive niches are marked by ECM stiffness and solid stress that limit drug delivery and immune-cell penetration [207,208]. Artificial intelligence-assisted approaches are emerging as potential tools to support the stratification of postoperative tumor states and the design of precision nanomedicine strategies. By integrating multimodal data, including medical imaging, clinicopathological characteristics, and molecular profiles, these models may enable a more comprehensive characterization of the postoperative microenvironment [209]. In this context, AI-based analyses have shown potential in estimating recurrence risk and identifying features associated with immune suppression or treatment resistance, which may help to refine patient selection for adjuvant therapies, including immunotherapy and nanomaterial-enabled interventions [210,211]. However, current applications remain largely exploratory and are constrained by data heterogeneity, limited cohort sizes, and a lack of standardized analytical frameworks. In addition, issues related to model interpretability and prospective validation need to be addressed before these approaches can be reliably translated into clinical decision-making. Therefore, AI-assisted prediction is expected to serve as a supportive framework for improving postoperative stratification and guiding treatment tailoring in precision nanomedicine. Emerging evidence further suggests that these stratified postoperative contexts may be associated with differential nanoplatform responses. For example, in highly immunosuppressive melanoma, ultrasmall nanoparticles activate cGAS–STING signaling and promote immune-mediated cell death, whereas ferroptosis may dominate in less suppressive or hypoxia-enriched niches [212]. Tumor type and stromal density also influence nanoparticle accumulation, indicating that ECM-modulating or penetration-enhancing designs may be required in dense or fibrotic niches [213], while environment-responsive platforms may help overcome metabolic and immune barriers [214]. Collectively, this stratification framework may guide the rational design of nanoplatforms and enhance their translational relevance in heterogeneous postoperative settings (Fig. 7A).

Second, the immunomodulatory effects mediated by nanomaterials still lack clear causal mechanistic resolution, which constrains rational design and optimization. Although many studies report that nanotherapeutic interventions suppress residual tumor recurrence by inducing ICD or remodeling the postoperative immune landscape, the underlying immune cascades remain incompletely defined [133,178]. In particular, the interactions of nanomaterials with immune cell subsets such as MDSCs [215], TAMs [216], and effector or memory T cells [217] are often described only at the endpoint, rather than tracked over time and linked to causality. This limitation is also reflected in the variable durability of therapeutic responses across residual tumor models. To address this issue, future studies should combine standardized perioperative sampling [218], selective immune cell depletion [219], and responder-versus-nonresponder comparisons [220]. These approaches may help identify the dominant cell populations, key signaling pathways, and critical temporal windows that determine efficacy. Such strategies would help move immuno-nanotherapeutic design from empirical combination toward more mechanism-driven integration with ICB, cancer vaccines, or other immunotherapies (Fig. 7B).

Third, insufficient evidence regarding long-term efficacy and biosafety remains a major barrier to clinical translation. Most studies are still confined to murine models with relatively short follow-up periods, leaving late recurrence, distant metastasis, chronic toxicity, and persistent immune sequelae insufficiently evaluated [221]. This limitation is particularly relevant for long-retained systems, such as hydrogels, implantable scaffolds, and inorganic nanoparticles. Although prolonged residence can enhance spatiotemporal control, it may also increase tissue exposure and long-term safety risks [222]. Delayed degradation or incomplete clearance can lead to sustained local accumulation [223], while prolonged or excessive inflammatory and complement activation may exacerbate immune-related adverse effects [224,225]. In addition, foreign body responses with fibrotic encapsulation may compromise local tissue compatibility [226]. and retained nanomaterials may introduce signal-related artifacts or other effects that complicate postoperative imaging assessment [227]. These considerations underscore the need to balance sustained local efficacy with biodegradability, clearance, and long-term biosafety. Accordingly, greater emphasis should be placed on the rational design of biodegradable and clearable systems [228,229], dose optimization based on safety-efficacy balance [230,231], and longitudinal in vivo monitoring of biodistribution, metabolism, and clearance [232,233]. Together with extended follow-up and more standardized evaluation of biocompatibility, stability, toxicity, and pharmacokinetics in orthotopic and large-animal models [234], these efforts may provide a more robust and clinically relevant framework for translating sustained local therapies while ensuring long-term safety (Fig. 7C).

Finally, the clinical translation of nanomaterial-based therapies for postoperative and post-ablation residual tumors remains constrained by challenges in standardization, manufacturing, regulation, and clinical implementation. Current residual tumor models and efficacy evaluation frameworks are not yet sufficiently standardized, limiting cross-study comparability and hindering objective assessment of therapeutic benefit [[235], [236], [237], [238]]. From a regulatory perspective, the European Medicines Agency (EMA) 2025 horizon-scanning report indicates that nanomedicines are not governed by a dedicated legal framework and highlights persistent challenges related to product classification, critical quality attributes, and the control of scale-up, stability, and impurities [239]. In the United States, multifunctional platforms that integrate drug, device, or biomaterial components may be regulated as combination products, introducing additional complexity, as compliance must be demonstrated under applicable frameworks, including 21 Code of Federal Regulations Part 4 [240]. Moreover, increasing structural and functional complexity often raises unresolved issues in manufacturability, batch-to-batch reproducibility, scalability, and cost, which may limit translational feasibility [241,242]. Importantly, successful clinical implementation will also depend on the ability of these systems to integrate into real-world clinical workflows, including intraoperative deployment, retention within postoperative cavities, and compatibility with existing surgical or ablation procedures. Addressing these interconnected challenges will be essential for advancing multifunctional nanoplatforms toward clinically approvable and implementable therapies (Fig. 7D).

Despite these challenges, nanomaterials remain highly promising for the management of postoperative and post-ablation residual tumors. Further progress will depend on strategies that improve precision, adaptability, and clinical implementability. In this context, AI-guided design frameworks may support more accurate stratification of postoperative tumor states and enable the rational optimization of local nanomedicine interventions. In parallel, the development of programmable immune biomaterials capable of modulating the postoperative microenvironment in a context-dependent manner represents a promising direction. In particular, smart nanomaterials that can adapt their therapeutic activity in response to dynamically evolving postoperative microenvironmental cues, together with real-time, closed-loop intraoperative or perioperative theranostic systems, may enable more precise spatial and temporal control of treatment. Collectively, these directions may help bridge the gap between mechanistic design and clinical implementation, thereby advancing more effective and individualized management of residual tumors.

## CRediT authorship contribution statement

**Meiyan Zou:** Visualization, Writing – original draft, Writing – review & editing. **Xu Chen:** Writing – original draft, Writing – review & editing. **Nina Li:** Visualization, Writing – original draft, Writing – review & editing. **Zihao Zhou:** Visualization, Writing – original draft, Writing – review & editing. **Weiyao Feng:** Visualization, Writing – original draft, Writing – review & editing. **Rongwei Xu:** Writing – original draft, Writing – review & editing. **Xinyuan Zhao:** Conceptualization, Funding acquisition, Investigation, Methodology, Project administration, Supervision, Validation, Writing – original draft, Writing – review & editing. **Li Cui:** Conceptualization, Funding acquisition, Investigation, Methodology, Project administration, Supervision, Validation, Writing – original draft, Writing – review & editing.

## Declaration of competing interest

The authors declare that they have no known competing financial interests or personal relationships that could have appeared to influence the work reported in this paper.

## Data Availability

No data was used for the research described in the article.
