# Supplementary material for: Biomaterial-based strategies for postoperative residual tumors: From margin clearance to immune control and tissue repair
Source: Mater Today Bio. 2026 May 2;38:103167. doi: 10.1016/j.mtbio.2026.103167 (PMC13158432; doi:10.1016/j.mtbio.2026.103167)
Supplement: Multimedia component 1 [file mmc1.docx]

**Biomaterial-based strategies for postoperative residual tumors: from margin clearance to immune control and tissue repair**

Meiyan Zou^a, #^, Xu Chen^a, #^, Nina Li^a^, Zihao Zhou^a^, Weiyao Feng^a^, Rongwei Xu^a^, Xinyuan Zhao^a, *^, Li Cui^a, b*^

^a^Stomatological Hospital, School of Stomatology, Southern Medical University, Guangzhou, 510280, Guangdong, China.

^b^School of Dentistry, University of California, Los Angeles, Los Angeles, 90095, CA, USA.

^#^Equal contributors

*Correspondence

Xinyuan Zhao, Email: zhaoxinyuan1989@smu.edu.cn

Li Cui, Email: zsucllj@ucla.edu

**Supplementary Table 1. Additional nanomaterial-enabled localized delivery platforms for residual tumor treatment.**

| **Nanomateial delivery system** | **Loaded drug** | **Release mechanism** | **Local delivery advantage** | **Tumor model** | **Therapeutic outcome** | **Ref.** |
| --- | --- | --- | --- | --- | --- | --- |
| NDP@MnO_2_ hydrogel | MnO_2_ | Thermo-responsive sol-gel transition | Adhering strongly to wet tissue and conforming to irregular wound surfaces | Residual pancreatic tumor after resection | Selectively eliminating residual tumor cells via combined PTT | [64] |
| Cur@HMON@gel | Cur | Sustained release with ultrasound-triggered SDT | Maintaining prolonged local drug exposure for over half a month | Residual RCC after incomplete TA | Promoting apoptosis and inhibiting proliferation of residual RCC cells | [65] |
| PVA-MoS_2_-R837 self-healing hydrogel | R837 | Diffusion-controlling sustained drug release | Remolding to fit tissue surfaces and adhering to surrounding tissue | Postoperative residual breast cancer | Suppressing postoperative recurrence via combined PTT and immunotherapy | [69] |
| DOXC_12_-LNC^CL^ hydrogel | DOXC_12_ | Diffusion- and degradation-mediated release | Providing durable local chemotherapy within resection sites | Postoperative orthotopic GBM | Delaying tumor recurrence and extending median survival to 42 d | [70] |
| PNP_PTX_&MNP_CpG_ hydrogel | PTX, CpG | Thermo-induced gelation–controlled release | Enhancing local brain delivery via bypassing the BBB | Postoperative orthotopic GBM | Killing residual glioma cells, activating anti-tumor immunity, prolonging median survival to 50 d | [73] |
| Thermoresponsive hydrogel co-delivering Cur nanomedicine and nanovaccine | Cur, antigenic peptide, CpG-ODN | Thermo-induced in situ gelation-controlled release | Enabling localized spatiotemporal co-delivery at the surgical bed | Postoperative residual breast cancer | Inducing durable systemic antitumor immunity, ↓84% recurrent tumor burden, ↓80.77% lung metastasis | [74] |
| TM@Gel | Celastrol loaded micelles (TM) | Releasing via ROS-responsive gel degradation | Conforming to wound cavity, scavenging ROS, improving TME | Postoperative residual breast cancer | 89.35% tumor suppression, inhibiting lung metastasis, improving TME | [75] |
| BSA/PTX NPs incorporated hydrogel_Gd_/EPI | EPI, PTX | Thermosensitive rapid gelation and staged dual-drug release | Enabling MRI tracking and rapid in situ solidification | Postoperative residual hypodermic brain tumor | Inhibiting tumor progression, recurrence-free ＞63 d, ↑median survival ＞63 d | [76] |
| PNP@(¹³¹I-Hyp) | ¹³¹I-Hypericin | Necrosis-targeted accumulation and radiotherapeutic activity | Preferentially depositing in ablation-induced necrotic region, enabling fluorescence and SPECT imaging, and prolonged tumor retention | Residual subcutaneous HCC after thermal ablation | 67.40% residual tumor suppression via necrosis-targeted radiotherapy with minimal off-target toxicity | [80] |
| DOX@MSCPs | DOX, celecoxib | Redox-responsive dual release | Enhancing tumor accumulation while inhibiting the COX-2/PGE_2_ axis | Subcutaneous HCC and orthotopic breast cancer | 73% (HepG2) and 91% (4T1) tumor suppression, ↓67% lung metastasis, reducing drug resistance, prolonging survival | [81] |
| LTSPN | HCPT | Lysosomal uptake, acid-triggered hydrolysis, and intracellular condensation into silicon nanoparticles | Targeting lysosomes, preventing lysosomal drug sequestration, enhancing intracellular retention | Post-resection residual bladder tumor | 62.70% residual tumor suppression, overcoming chemoresistance | [82] |
| PMVS-P | SAL | Targeted delivery via D2 dopamine receptor–mediated uptake | Conforming to surgical cavity and targeting residual GSCs | Postsurgical residual GBM | 91.76% inhibition of GBM recurrence at day 25 by eliminating GSCs | [86] |
| NM-PD | DOX | Inflammatory chemotaxis-mediated targeting | Targeting postsurgical cavity and crossing inflamed BBB | Postsurgical residual GBM | ↓85.12% residual tumor proliferation, ↑median survival to 37 d | [87] |
| BP@DOX/PEG-FA-GP composite scaffold | DOX | NIR-triggering sol–gel transition and degradation-mediated release | Combining photothermal-chemotherapy, promoting tissue repair via ERK1/2 and PI3K/Akt activation | Subcutaneous melanoma with wound healing | 82.86% tumor inhibition, nearly 100% wound healing at day 15 | [159] |

**Supplementary Table 2. Combinatorial therapeutic strategies enabled by nanomaterials against residual tumors.**

| **Nanoplatform** | **Synergistic modality** | **Mechanism** | **Tumor model** | **Synergistic efficacy** | **Ref.** |
| --- | --- | --- | --- | --- | --- |
| Trojan bacteria | PTT | Releasing tumor‑associated antigens and PAMPs, activating innate and adaptive immunity | Orthotopic glioblastoma | 70.11% (Trojan VNP) and 66.25% (Trojan EC) tumor destruction, stimulating immune response, prolonging survival | [92] |
| CuS@MSN-TAT-RGD NPs | Nuclear-targeted PTT | Targeting tumor cell nuclei, inducing exhaustive apoptosis | Postoperative residual cervical cancer | 0% recurrence at day 20, achieving long‑term tumor control | [93] |
| FIP | PTT | Inducing ICD and blocking ATP-adenosine pathway-mediated immunosuppression | Residual breast cancer after incomplete PTT | 80% survival at day 45, establishing immune memory, ↑response to PD-1 blockade | [97] |
| JQ-1@PSNs-R | PTT | Enhancing PTA while inhibiting BRD4-driven tumor progression, with antitumor immune activation | Residual melanoma after incomplete PTT | Eradicating residual tumor cells, 66.67% tumor-free over 50 days | [98] |
| VI@Gd-NPs | PTT + Gd-enhanced MRI | Disrupting tumor vasculature, enhancing heat deposition, releasing in-situ tumor vaccine | Residual CRC after incomplete PTT | ↑antitumor immune responses, 69% survival ＞100 d, ↓100% rechallenged tumor | [99] |
| CCP NPs | LTPTT + PDT | Targeting tumor cell membranes to induce ICD and activate antitumor immunity | Breast cancer | 91.88% tumor suppression, 0/5 lung metastasis, 60% survival at day 60 | [106] |
| Ru/GOx@Hydrogel | PDT + PTT + starvation therapy | Generating ROS while consuming glucose to synergistically killing residual tumor cells | Melanoma with surgical wound | Complete residual tumor eradication and wound healing at day 14 | [107] |
| DDTB-based nanoparticles | NIR fluorescence imaging + PDT + PTT | Enabling fluorescence-guided resection and eradicating microscopic residual tumors via PTA and photodynamic ablation | Postoperative residual cervical tumor | ↓90% recurrence, ↑median survival >70 d, with further enhancing PD-L1 blockade efficacy | [108] |
| PWCu nanocapsules | Radiotherapy | Triggering radiation‑induced cuproptosis and activating systemic immunity | Radioresistant and re‑irradiation breast tumor | ↑median survival to 90 d, 40% complete response to radiotherapy | [111] |
| Fe-TPZ NPs | RFA | Inducing lipid peroxidation via Fenton reactions, triggering tumor cell death and promoting antitumor immune responses | Subcutaneous liver cancer and orthotopic breast cancer after iRFA | Inhibiting tumor growth, prolonging survival | [112] |
| P@Fe SAZ | Radiofrequency dynamic therapy with identical trigger | Generating ROS under radiofrequency stimulation and remodeling immunosuppressive TME | Residual subcutaneous HCC after iRFA | Suppressing residual tumor growth, preventing tumor relapse and lung metastasis, prolonging survival | [114] |
| Mitochondrial-targeted Ca/Cu dual-ion chaos inducer | Cuproptosis + PTT | Disrupting mitochondrial ion homeostasis and inducing calcium overload to amplify cuproptosis while enhancing immune activation | Postoperative residual melanoma | Improving melanoma suppression, achieving 89.7% wound healing at day 12 | [115] |
| GOx@MnCaP@fibrin | Starvation therapy + CDT | Depleting glucose and generating ROS via Fenton-like reactions, inducing tumor cell death | Postsurgical residual IDH1 (R132H) glioma | ↓62.5% residual glioma recurrence, 82.5% survival at day 60 | [117] |
| GA@CaMP hydrogel | Ultrasound | Inducing apoptosis, remodeling immune microenvironment, promoting osteogenesis | Postoperative residual breast cancer | Inhibiting tumor recurrence, 87.5% survival at day 40, accelerating bone defect repair with 30.97% BV/TV at 8 week | [120] |

**Supplementary Table 3. Extended immunomodulatory strategies of nanomaterials for residual tumor control.**

| **Nanomaterial** | **Immunomodulator** | **Immune effect** | **Tumor model** | **Immune outcome** | **Ref.** |
| --- | --- | --- | --- | --- | --- |
| P-DOX/1MT@MM-Gel | 1-MT | Inducing ICD, promoting DC maturation; increasing tumor-infiltrating CTLs | Residual melanoma after resection | 43.82% tumor suppression, reducing tumor recurrence | [124] |
| DTX-loaded Zein/CSP-GTP/Fe^III^ NPs | CSP | Inducing chemo-photo-immune-amplified ICD, facilitating DC maturation, enhancing T cell infiltration | Subcutaneous lung and breast tumor | 100% primary and distant tumor eradication, 100% survival at day 30 | [126] |
| Gel-Fe-PA | Photothermal immunomodulation via ICD | Inducing photothermal ICD, releasing DAMPs, activating DCs | Bilateral subcutaneous breast tumor | Activating systemic antitumor immunity, suppressing local and distant tumor growth | [127] |
| BMCPH | l-cysteine–derived H_2_S | Inhibiting MDSC accumulation and enhancing CTL infiltration | Residual breast tumor after thermal ablation | Reducing recurrence and metastasis via immune reactivation | [130] |
| man-IONPs | d-mannose | Reprogramming M2 macrophages toward M1 phenotype | Residual orthotopic HCC after iMWA | Alleviating local immunosuppression, 88% tumor inhibition | [131] |
| ICG-SB@Lip-ZA nanosystem | ZA, SB-505124 | Reducing M2 macrophages and suppressing CAF-mediated immunosuppression to increase intratumoral T cell infiltration | Residual subcutaneous breast cancer after PTT | Achieving robust tumor eradication, enhancing antitumor immunity | [132] |
| MSA-2-loaded EXOs incorporated within MN patches (MEM) | MSA-2 | Activating STING signaling, increasing IFN and promoting DC maturation | Residual subcutaneous breast tumor after FLASH radiotherapy | Enhancing antitumor immunity, 89.1% residual tumor suppression, inhibiting metastasis | [134] |
| THINR-CXCL10@Gel | MIT, siIDO1, CXCL10 | Inducing ICD, enhancing T cell recruitment and relieving Treg-mediated immunosuppression | Postsurgical residual GBM | Suppressing postoperative recurrence via sustained immune activation | [135] |
| TPA-FFG-LA | TPA | Inducing ICD via ROS and lysosomal damage | Residual TNBC after PTT | Eliminating residual tumor cells, suppressing tumor growth | [136] |
| P-P-IO | aPD-L1, IONPs | Inducing photothermal-mediated ICD, activating T cells | Residual postsurgical breast tumor | 66.7% recurrence suppression through reinforced antitumor immunity | [142] |
| IPI549@HMP | IPI549 | Inducing hypoxia‑relieved ICD, reprogramming immunosuppressive TME; activating DCs and CTLs | Postsurgical residual colon tumor | Suppressing residual tumor growth, 50% complete response to RT, 100% survival at day 60 | [144] |
| PAG nanofiber patch | Anti–PD-L1 antibody | Inducing ICD, promoting DC maturation and enhancing effector T cell infiltration | Postsurgical residual HCC | 66.7% recurrence suppression, ↓metastasis, ↑survival; ↑PD‑L1 antibody efficacy | [145] |
